# High-dimensional immune profiling of follicular fluid and systemic circulation reveals distinct immune signatures in women with polycystic ovary syndrome

**DOI:** 10.3389/fimmu.2025.1628031

**Published:** 2025-08-11

**Authors:** Soma Banerjee, Fernanda L. Jaimes, Mona A. Mohamed, Abigail Zettel, Nesya G. Graupe, Laura G. Cooney, Aleksandar K. Stanic

**Affiliations:** ^1^ Division of Reproductive Sciences, Department of Obstetrics and Gynecology University of Wisconsin, Madison, WI, United States; ^2^ Division of Reproductive Endocrinology and Infertility, Department of Obstetrics and Gynecology, University of Wisconsin, Madison, WI, United States

**Keywords:** polycystic ovary syndrome, immune cells, cytokines, high-dimensional flowcytometry, *in-vitro* fertilization polycystic ovary syndrome, *in-vitro* fertilization

## Abstract

**Background:**

Persistent low-grade inflammation has been hypothesized as a possible key contributor to polycystic ovary syndrome pathophysiology through associative studies. Since immune cells within the ovarian follicle—the central site of PCOS dysfunction—play pivotal roles in immune defense and regulation of ovulation, establishing a definitive cellular map of normal and PCOS-affected follicular immune composition is essential.

**Method:**

This is a prospective cohort study of women with PCOS (Rotterdam criteria) and controls undergoing *in vitro* fertilization (IVF). Peripheral blood was collected before treatment (visit 1) and again at transvaginal oocyte retrieval (TVOR, visit 2). Follicular fluid (FF) was obtained from the first two dominant follicles during TVOR. We measured the cytokines and angiogenic factors in both plasma and FF using multiplexed cytometric bead assays. The cellular immune composition was evaluated by using high-dimensional multispectral flow cytometry, followed by dimensionality reduction and graph-based clustering analyses.

**Results:**

We found that the TVOR plasma contained significantly higher concentrations of IL-2, IL-4, IL-9, IL-17A, TNF-α, and MCP-1 compared to the follicular fluid, whereas the follicular fluid was enriched with angiogenic factors such as VEGF and EGF. Notably, pre-treatment plasma samples from PCOS patients showed elevated Il-4, IL-6, IL-9, and IL-10, which were partially resolved by TVOR. Moreover, the PCOS follicular fluid exhibited higher numbers of classical monocytes and a trend toward increased CTLA4-positive T regulatory cells relative to the controls.

**Conclusion:**

Our findings highlight a compartment-specific immunome in PCOS, marked by distinct cytokine and angiogenic factor distributions in circulation versus follicular fluid. PCOS was characterized by elevated systemic inflammatory markers before treatment, which were partially normalized by TVOR, yet key immune differences persisted at the follicular level. These results underscore the utility of comprehensive multiparametric analyses—including high-dimensional flow cytometry—to uncover immune dysregulation and identify potential therapeutic targets in PCOS.

## 1 Introduction

Polycystic ovary syndrome is the most common endocrine disorder in women of reproductive age with a prevalence of 10% worldwide (1, 2). PCOS is defined by the Rotterdam diagnostic criteria (2003), and the 2023 International PCOS Guidelines defines PCOS as the presence of two out of three of the following: (1) clinical or biochemical hyperandrogenism, (2) oligomenorrhea, and (3) polycystic ovaries on ultrasound. There is an increased risk to reproductive, metabolic, cardiovascular, and psychological health (3–6). Accumulating evidence indicates that PCOS is not merely an endocrine disorder but may also involve persistent low-grade inflammation (a hypothesis under current investigation) and associated metabolic disturbances such as obesity, insulin resistance, and type 2 diabetes (7–10). Elevated proinflammatory cytokine markers (11–19) and altered immune populations (20, 21) in PCOS suggest that immune factors may contribute to ovulatory dysfunction. Furthermore, normal ovulatory processes rely on immune cells and cytokines to regulate follicle development, oocyte maturation, follicular rupture, and corpus luteum formation (22–31). Therefore, clarifying the interplay between these immune mechanisms at the ovarian follicle and systemic levels is essential to understanding PCOS pathogenesis. However, it remains unclear to what extent systemic immune changes reflect the ovarian immune environment, a gap that the present study aims to address by directly comparing circulating versus follicular immune profiles in PCOS.

The systemic/circulating proinflammatory milieu in PCOS is suggested in several studies, including elevated plasma levels of C-reactive protein (CRP) (11, 13, 18, 19), interleukin 18 (IL-18) (14, 16), monocyte chemoattractant protein-1 (MCP-1) (17, 32), and macrophage inflammatory protein-1α (MIP-1α) (17, 33). The precise cellular origins of these circulating cytokines are currently unknown. Notably, only a few studies focused on understanding circulatory immune cellular changes, demonstrating an elevation of circulatory total leukocyte counts and immune cell subsets (lymphocytes, neutrophils, NK cells) in PCOS (20, 21). Initial studies, which employed follicular fluid isolated at the time of transvaginal oocyte retrieval (TVOR) in *in vitro* fertilization (IVF), suggest higher levels of pro-inflammatory markers (TNF-α, CRP, IL-6, IL-2, and IFN-γ) in PCOS (34–36). However, these earlier studies targeted only a limited range of immune mediators and lacked a comprehensive, high-dimensional analysis of both cytokine expression and cellular composition. Furthermore, the populations previously studied are largely ethnically homogenous and not broadly reflective of the US, European, and worldwide PCOS burden in characteristics such as BMI and metabolic abnormality (16, 37–39).

Cellular and cytokine alterations are significant because normal immune system function is necessary for successful ovulation (40, 41). Within the ovary, immune cells secrete local cytokine mediators that promote follicle development, oocyte maturation, timely rupture of follicles, angiogenesis, corpus luteum development, and luteal demise (23–31), all of which may be altered in PCOS and which may contribute to or be a consequence of this disease.

Despite evidence of inflammatory abnormalities in PCOS, no single study has simultaneously characterized the local (follicular fluid) and systemic (circulatory) immunome at high resolution to reveal clinically relevant immune alterations. Furthermore, immune cells resident in organ stroma are composed of different subsets than those in circulation, as befits their role in regulating organ-specific immunity. Whether ovarian follicles during maturation and ovulation also have a distinct composition compared with the systemic circulating compartment is not well understood, so we assessed it here in detail. By profiling both immune compartments in parallel, we also aimed to determine whether peripheral immune parameters accurately reflect local ovarian immune changes in PCOS. Although individual cytokine and cellular assessment can suggest valuable targets for larger studies and mechanistic investigations, it is increasingly clear that comprehensive, multi-modality assessments are necessary to determine clinically relevant and actionable immune targets. High-dimensional approaches that investigate immune dysfunction in PCOS at several scales and locations (cytokine, angiogenic growth factors, and immune cells in both circulation and follicles) can yield a novel and comprehensive atlas of systemic and follicular immune signature. This information will provide leverage to mechanistically dissect immune pathways dysregulated and determine the components of this phenotype in PCOS.

## 2 Methods

### 2.1 Human samples

This is a prospective cohort study of women with PCOS (Rotterdam criteria) and controls at an academic infertility clinic, as approved by the University of Wisconsin-Madison IRB protocol (#2019-1247). The inclusion criteria for the PCOS group were defined as those satisfying the Rotterdam criteria (2). The control inclusion criteria include regular menstrual cycles and normal ovarian reserve (anti-Mullerian hormone (AMH) >1 ng/mL or antral follicle count (AFC) >10). The control individuals also have a fertility diagnosis that includes either male factor infertility (defined as a semen analysis with <15 million sperm/mL or <15 million motile sperm/ejaculate), tubal factor infertility (defined as no patent Fallopian tubes), or use of preimplantation genetic testing (PGT-M) for an inherited condition. The exclusion criteria for both groups are current pregnancy, endometriosis (any stage), type 1 diabetes, immuno-modulating medications, autoimmune conditions, active malignancy, recurrent pregnancy loss (two prior pregnancy losses after clinical detection of intrauterine pregnancy), *in situ* hydrosalpinx without ligation, and age <18 or >44 years.

All participants underwent controlled ovarian stimulation using either a standard luteal Lupron overlap or GnRH antagonist protocol, stimulated with individualized doses of recombinant FSH and purified human menopausal gonadotropins, with the final oocyte maturation triggered 36 h before transvaginal oocyte retrieval by either full-dose hCG (10,000 units) or a Lupron trigger supplemented with low-dose hCG (1,000 units).

Peripheral blood was collected at pre-treatment (visit 1) and at transvaginal oocyte retrieval (TVOR, visit 2), as well as follicular fluid (FF) at visit 2 retrieved from two dominant follicles, with care taken to avoid blood contamination. We have matched the visits 1 and 2 and FF samples for 30 patients (10 control and 20 PCOS) for the cytokine studies and also matched the visits 1 and 2 and FF samples for 33 patients (13 control and 20 PCOS) for the flow cytometry studies. Note that the 33 matched cellular samples include all 30 processed for cytokines (**Table 1**). The follicular fluid aspirates in oocyte isolation dishes were serially examined, and those with visible blood contamination were discarded. In this study, none of the analyzed FF samples had significant blood contamination.

**Table 1:** Clinical characteristics of the study subjects.

Variable	All subjects (*N* = 33)	Control (*n* = 13)	PCOS (*n* = 20)	*P*-value
Medical history				
Age (years)^a^	31.8 ± 3.3	31.8 ± 3.6	31.8 ± 3.2	0.9
BMI (kg/m^2^)^a^	30.0 ± 6.9	28.1 ± 6.6	31.2 ± 6.9	0.2
Diabetes	2 (6.1)	0	2 (6.1)	0.5
Hypertension	3 (9.1)	1 (7.7)	2 (10.0)	1.0
Depression	5 (15.2)	4 (30.1)	1 (5)	0.07
Anxiety	7 (21.2)	3 (23.1)	4 (20.0)	1.0
Fertility history				
Nulliparous	14 (42.4)	2 (15.4)	12 (60.0)	0.02
Length of infertility (months)^b^	24 (17, 36)	24 (15, 30)	27 (17.5, 36)	0.5
AMH^b^	4.6 (3.0, 6.4)	3.4 (1.6, 4.2)	6.2 (4.4, 11.7)	0.001
PCOS-specific variables		Not applicable		
Oligomenorrhea			18 (90%)	
Hyperandrogenism			12 (60%)	
Polycystic ovarian morphology			18 (90%)	

Categorical variables are reported as *n* (%). Values are mean ± SD. *P*-values from unpaired *t*-tests comparing PCOS vs. control.

a Continuous variables reported as mean and standard deviation.

b Continuous variables reported as median (interquartile range).

### 2.2 Sample collection and storage

Peripheral blood mononuclear cells (PBMC) were separated by density gradient centrifugation and cryopreserved in liquid nitrogen storage, along with the cell pellet from follicular fluid. Supernatant plasma and follicular fluid were stored at -80°C until the experiments were conducted.

### 2.3 Cytometric bead assay for cytokines

The plasma and follicular fluid cytokine levels of 33 analytes (**Table 2**), including adaptive helper cytokines, innate response cytokines, angiogenesis, and growth factors, were determined by employing multiplexed, custom cytometric bead arrays (LEGEND Plex, Biolegend, San Diego, CA, USA) according to the manufacturer’s instruction.

**Table 2:** List of analytes investigated by using LegendPlex.

Adaptive helper cytokines	Innate response cytokines	Angiogenesis and growth factors
IL-2	IL-1α	TGFβ
IL-4	IL-11	Angiopoietin 1
IL-5	IL-1β	Angiopoietin 2
IL-6	IL-15	EGF
IL-9	GM-CSF	PIGF
IL-10	IL-18	VEGF
IL-13	IL-23	MCP-1
IL-17A	IL-33	TSLP
IL-17F	IFNα2	
IL-21	IL-12p40	
IL-22	IL-12p70	
IFNγ	IL-27	
TNFα		

### 2.4 Flow cytometry labeling and analysis

Frozen PBMC and follicular fluid were thawed, and cell suspensions were first labeled with LIVE/DEAD^®^ fixable blue stain (Invitrogen, cat. no. L34962) for 10 min, followed subsequently by staining with three separate cocktails of 26–28 fluorochrome conjugated monoclonal antibodies (**Table 3**). The antibodies were diluted in BD Horizon BrilliantTM Stain Buffer (BD Biosciences, San Jose, CA, USA, cat. no. 566349) and used to label cells for 30 min, washed, and fixed with 4% formaldehyde (Ted Pella Inc., Redding CA, USA, cat. no. 1805) for 5 min before washing with stain buffer (BD, cat. no. 554656). Transcription factor assessment was done by overnight permeabilization of cells using BD Pharminogen™ Transcription Factor Buffer Set (BD Biosciences, cat. no. 562574), followed by intracellular antibody labeling for 50 min. All antibody incubations were performed at 4°C–8°C. Appropriate single-stained controls are used on cells and/or UltraComp eBeads (eBioscience, cat. no.01-222-42) and fluorescence minus one (FMO) controls on cells. Multispectral flow cytometry was performed using a five-laser spectral cytometer Aurora (Cytek Biosciences, Fremont, CA, USA).

**Table 3:** Flow cytometry antibodies.

Marker	Fluorophore	Catalog no.	Clone	Vendor	Volume, µL/100 µL
CD45 RA	BUV395	740315	5H9	BD Biosciences	0.25
CD45	BUV496	750179	HI30	BD Biosciences	0.25
CD14	BUV563	741441	MϕPg	BD Biosciences	0.5
CD11c	BUV661	612967	B-Ly6	BD Biosciences	0.5
CD56	BUV737	612766	NCAM16.2	BD Biosciences	0.5
CD45 RO	BUV805	748367	UCHL1	BD Biosciences	0.5
CD141	BV421	344114	M80	Biolegend	0.5
CD161	eFlour450	48-1619-42	HP3G10	Invitrogen	0.5
CD11b	BV480	746572	D12	BD Biosciences	0.5
CD4	BV510	300546	RPA-T4	Biolegend	0.5
HLA DR	BV570	307637	L243	Biolegend	0.5
CD25	BV605	562660	2A3	BD Biosciences	0.5
CCR6/CD196	BV711	353436	G034E3	Biolegend	0.5
CD127	BV785	351330	A019D5	Biolegend	0.5
CD117	BB515	565172	104D2	BD Biosciences	0.5
CD8a	SparklingBlue550	344759	5K1	Biolegend	0.25
CD94	PerCP Cy 5.5	562361	HP-3D9	BD Biosciences	0.5
EOMES	PE eFluor610	61-4877-42	WD1928	Invitrogen	2
CD16	NovaFluorYellow700	H006T03Y07	348	Invitrogen	0.5
CD49a	PE-Vio770	130-101-403		Miltenyl Biotech	0.5
CD49a	PE-Cy7	328312	TS2/7	Biolegend	0.5
RORyT	APC	17-6988-82	AFKJS-9	Invitrogen	2
CCR7	SparklingNIR685	353257	G043H7	Biolegend	0.5
CD123	R718	752032	9F5	BD Biosciences	0.5
CD19	APC Fire750	363030	SJ25C1	Biolegend	0.5
CD3	APC Fire810	344858	SK7	Biolegend	0.5
FOXP3	PE	12-4776-42	PCH101	Invitrogen	2
Helios	PE Cy5	15-9883-42	22F6	Invitrogen	2
Tbet	PE	12-5825-82	eBio4B10	Invitrogen	2
CD69	BV650	310934	FN50	Biolegend	0.5
LILRB1	PE-eFluor 610	61-5129-42	HP-F1	Invitrogen	0.5
NKG2A	APC	375108	S19004C	Biolegend	0.5
CD279/PD-1	PE-eFluor 610,	61-5129-42	eBioJ105	Invitrogen	0.5
CTLA4	APC	369612	BNI3	Biolegend	0.5
CD103	PE	12-1038-42	B-Ly7	Invitrogen	0.5
CD39	PerCP Cy 5.5	328218	A1	Biolegend	0.5

### 2.5 Data analysis

LEGENDplex™ complimentary cloud-based software was used to quantify cytokines. Manual data analysis was performed using JMP Student Edition v.18 (JMP Statistical Discovery LLC, Cary, NC, USA) and Prism^®^ v. 9.4.1 (GraphPad Software, Inc., La Jolla, CA, USA). We compared the differences between plasma vs. follicular fluid (paired samples) and control vs. PCOS (independent groups) with Student’s *t*-test as appropriate. Spectral flow cytometry data was unmixed with SpectroFlo^®^ v 2.1 (Cytek Biosciences), followed by manual analysis of unmixed files with FlowJo v.10.8.1 software (FlowJo LLC, Ashland, OR, USA) and statistical graphing by using JMP Student Edition v.18 and GraphPad Prism^®^ v. 9.4.1.

Dimensionality reduction was performed by using Barnes Hut-modified t-distributed stochastic neighbor embedding (t-SNE) in FlowJo. After manual operator-driven gating to exclude dead cells and irrelevant populations, each patient FCS data file was concatenated at equivalent cell analysis nodes into a single combined data file. Innate lymphoid cells (ILC) were concatenated at lineage negative (CD45+CD3-CD19-CD14-), T cell and APC were concatenated at parent CD45+ cellular node, and corresponding samples were aggregated. To avoid batch effects, all samples acquired in the same experiment were analyzed together in a single instantiation of tSNE. Cluster frequencies and mean fluorescence intensity (MFI) values were then calculated with FlowSom plugin within FlowJo.

We considered a two-way ANOVA to simultaneously assess the effects of sample compartment (plasma vs. FF) and disease status. However, preliminary analyses with a mixed-effects model indicated that there was no effect of either PCOS status or any significant interaction between PCOS status and compartment for the cytokine or cell population data. Therefore, we proceeded with simpler paired (within-subject) and unpaired (between-group) comparisons as appropriate to samples. All statistical tests were two-tailed, and *p <*0.05 was considered significant.

## 3 Results

### 3.1 Cytokine and angiogenic factors are distinct in circulation and follicular fluid

**Figure 1:**
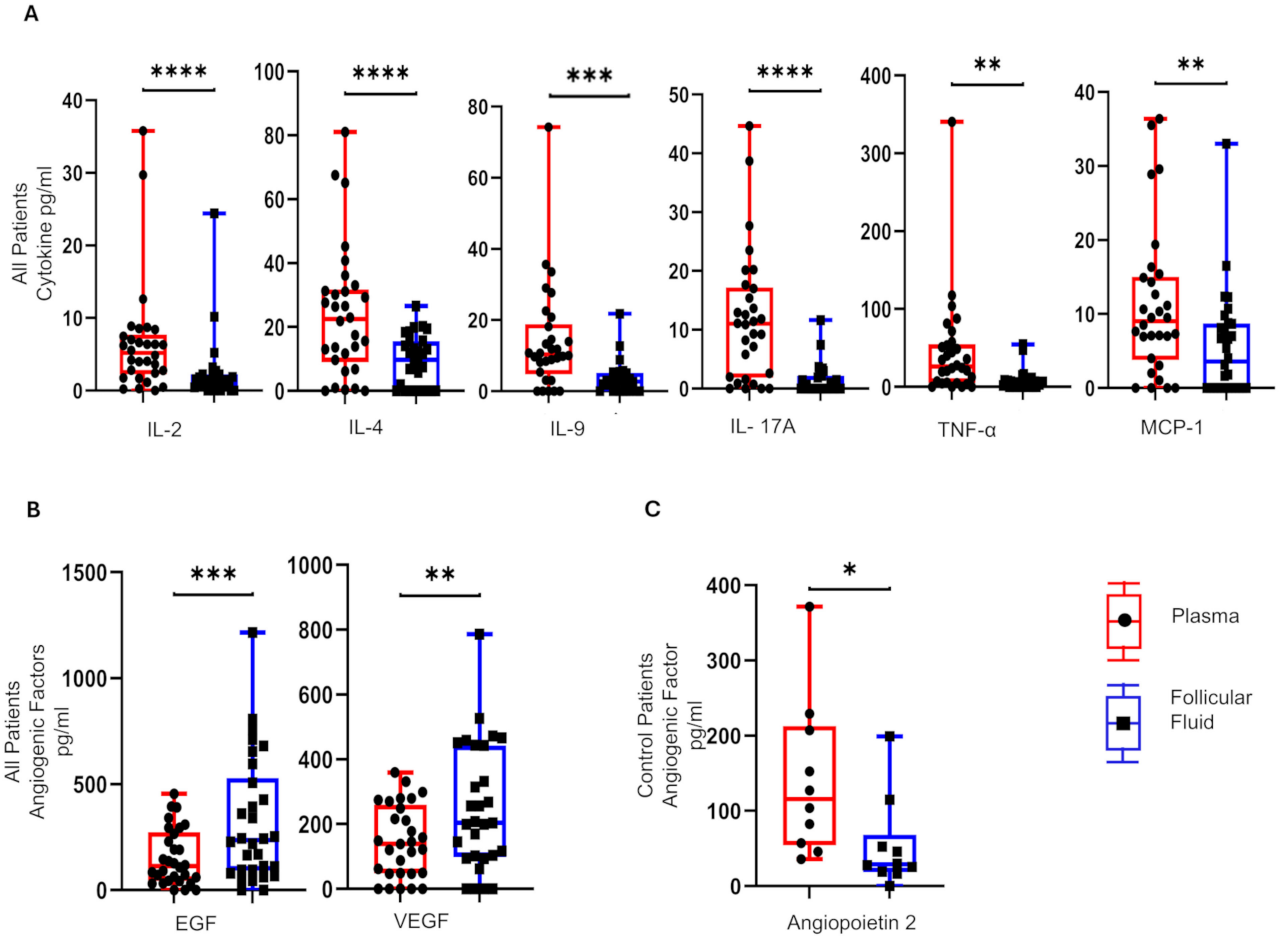
Differential distribution of cytokines and angiogenic mediators in plasma and matched follicular fluid. **(A)** IL-4, IL-2, IL-9, TNF-α, IL-17A, and MCP-1 are higher in plasma compared to follicular fluid in control and PCOS. **(B)** EGF and VEGF are higher in follicular fluid than plasma in control and PCOS. **(C)** The angiopoietin 2 levels were significantly higher in plasma in control compared to PCOS at TVOR. **(A, B)** combine PCOS and control groups (no interaction by disease status) and **(C)** only controls. *p <0.05, **p <0.005, ***p <0.0005, ****p <0.0001. *n* = 30, control = 10, PCOS = 20. Paired *t*-tests were used for plasma vs. FF comparisons. Only significant differences are shown.

To determine if the focal point of PCOS dysfunction, the ovarian follicle, has a distinct cytokine/angiogenic signature compared with circulating plasma, we determined the concentrations of 33 soluble factors in paired plasma and FF samples at the time of TVOR in all patients (*n* = 30, control = 10, PCOS = 20). The results revealed that IL-2, IL-4, IL-9, IL-17A, TNF-α, and MCP-1 in TVOR plasma were significantly higher (*p* < 0.05–0.0001) than in follicular fluid (**Figure 1A**). In contrast, the follicular fluid demonstrated increased angiogenic factors with the levels of VEGF and EGF being significantly higher (*p* < 0.05–0.001) than those of plasma (**Figure 1B**). Interestingly, plasma angiopoietin 2 was significantly higher than follicular fluid in the control group alone at TVOR (**Figure 1C**). Taken together, the results indicated that cytokine (immune) and angiogenic factors are divergently regulated in the ovarian follicle and systemic circulation. Notably, these differences were consistent in both PCOS and control groups, with no significant interaction by disease status. The plasma–FF differences were observed in both PCOS and control women without a significant PCOS-by-compartment interaction, suggesting that the compartmental cytokine profile reflects normal physiology rather than disease-specific changes.

### 3.2 Intrafollicular inflammatory compartment is distinct from circulation with differential distribution of key immune cell subsets

**Figure 2:**
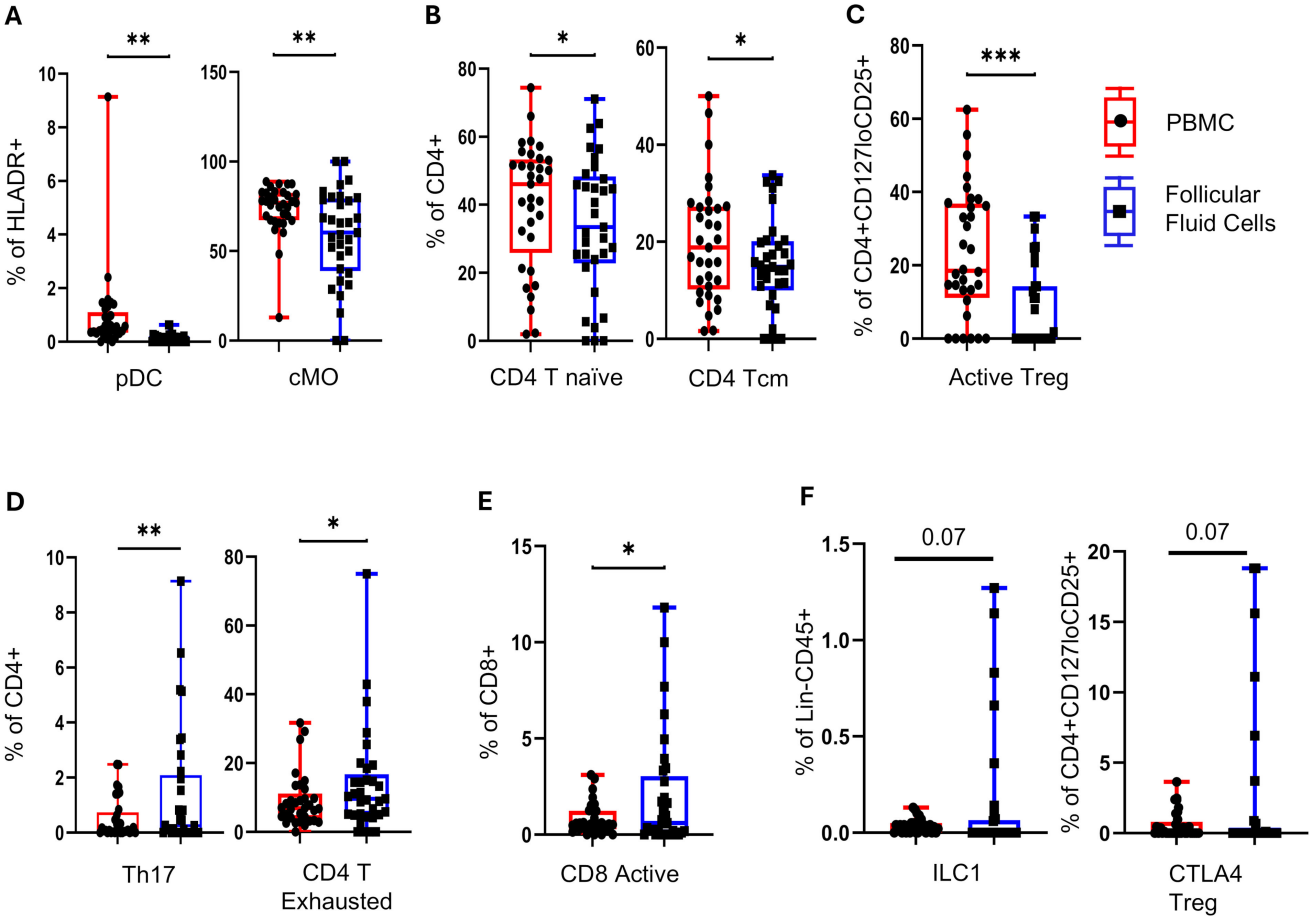
Follicle-specific immune cell composition at TVOR. **(A)** Plasmacytoid dendritic cells (pDC) and classical monocytes (cMO) are lower in follicular fluid than in blood. **(B)** CD4 T naïve and CD4 T central memory cells are lower in follicular fluid than in blood. **(C)** Follicular fluid has higher activated Treg cells. **(D)** In addition, follicular fluid has higher Th17 and CD4 T exhausted cells. **(E)** Follicular fluid has higher CD8 active cells. **(F)** Follicular fluid shows trends of higher innate lymphocytes: ILC1s and CTLA4 Treg cells are observed, though they are not statistically significant. *p <0.05, **p <0.05, ***p <0.005, ****p <0.0001. *n* = 33, control = 13, PCOS = 20. Significant differences and trends (0.05 < *p* < 0.1) are shown. Data in **(A–F)** combine PCOS and control groups (no interaction by disease status). Paired *t*-tests were used for plasma vs. FF comparisons.

As cytokine and angiogenic factors are produced by both the ovarian (local) and systemic circulating immune cells, we next determined the distribution of most major immune cell subsets in plasma and follicular fluid in all patients (*n* = 33, control = 13, PCOS = 20) as collected at the TVOR appointment. To accomplish this, we employed three high-dimensional flow cytometry panels using 26–28 distinct surface and intracellular makers for cell subset identification (**Table 3**). The complete gating strategy is explained in **Table 4** and **Supplementary Figures S1–S3**. We focused on the subsets of the overall antigen-presenting cells (**Figure 2A**, **Supplementary Figure S1**), adaptive immune/T cell compartment (**Figures 2B, D, E**, **Supplementary Figures S2B, C**), T regulatory (Treg) subsets (**Figures 2C, F**, **Supplementary Figure S2D**), and the innate immune compartment (**Figure 2F**, **Supplementary Figure S3**).

**Table 4:** Complete gating strategy by phenotype markers of flow cytometry experiments.

Cell type	Phenotype	Cell name
Innate lymphoid cells (ILCs) Lineage negative (Lin-) Lineage consists of CD3, CD14, and CD19	Lin-CD45+CD94-CD56+CD117+CD127+ Tbet+	ILC1
	Lin-CD45+CD56-CD94-CD117+CD127+RORyT+	LTi
	Lin-CD45+CD56+CD94-CD117+CD127+ RORyT+	ILC3
	Lin-CD45+ CD117-CD127- CD94+ CD16+CD56+ NK cells are of two types based on CD56 and CD16 expression level	NK cells
		CD56 bright
		CD56dim
Antigen-presenting cells gated for CD19, CD3, and CD56 negative. CD45+HLADR+	CD19-CD3-CD56-CD45+HLADR+CD14-CD16-CD11c-CD123+	Plasmacytoid dendritic cells (pDC)
	CD19-CD3-CD56-CD45+HLADR+CD14-CD16-CD123-CD11c+CD141+	Myeloid dendritic cells (mDC)
	CD19-CD3-CD56-CD45+HLADR+CD14+CD16+	Intermediate monocytes (iMO)
	CD19-CD3-CD56-CD45+HLADR+CD14-CD16+	Non-classical monocytes (ncMO)
	CD19-CD3-CD56-CD45+HLADR+CD14+CD16-	Classical monocytes (cMO)
T cells CD4 T cells CD19-CD3+CD4+	CD3+CD4+CCR6+CD161+	Th17
	CD3+CD4+CCR7-CD45RA+	CD4 T effector (eff)
	CD3+CD4+CCR7+CD45RA+	CD4 T näive
	CD3+CD4+CCR7+CD45RA-	CD4 T central memory (CM)
	CD3+CD4+CCR7-CD45RA-	CD4 T effector memory (EM)
	CD3+CD4+CD69+	CD4 active
	CD3+CD4+PD-1 +	CD4 exhausted
CD8 T cells CD19-CD3+CD8+	CD3+CD8+CCR7-CD45RA+	CD8 T effector (eff)
	CD3+CD8+CCR7+CD45RA+	CD8 näive
	CD3+CD8+CCR7+CD45RA-	CD8 T central memory (CM)
	CD3+CD8+CCR7-CD45RA-	CD8 T effector memory (EM)
	CD3+CD8+CCR7-CD45RA+LILRB1+	CD8 T effector memory RA (TEMRA)
	CD3+CD8+CD69+	CD8 active
	CD3+CD8+PD-1 +	CD8 exhausted
CD4 regulatory T cells Treg cells. CD4+ cells with CD25+CD127loFOXP3+	CD3+CD4+ CD25+CD127loFOXP3+	T regulatory (Treg)
	CD3+CD4+ CD25+CD127loCD45RA+	Näive Treg cells
	CD3+CD4+ CD25+CD127loCD45RO+.	Memory Tregs
	CD3+CD4+ CD25+CD127loHLADR+	Activated Tregs
	Helios, CTLA4, CD39	Other Treg markers

The plasmacytoid dendritic cells and classical monocyte are lower (*p* < 0.05) in follicular fluid (**Figure 2A**). The follicular fluid had a lower number of CD4 T naïve and CD4 T central memory cells (**Figure 2B**) and activated Treg cells (**Figure 2C**) (*p* < 0.05). In contrast, the follicular fluid had higher Th17 and CD4 T exhausted T cells (*p* < 0.05) (**Figure 2D**) and activated CD8 T cells (**Figure 2E**). Trends in follicular fluid enrichment of innate lymphocytes ILC1s and CTLA4 Treg cells are observed, though they do not reach statistical significance (0.05 < *p <*0.1) (**Figure 2F**). Taken together, these results indicate that follicular fluid maintains a distinct immune cellular composition, with a bias toward differentiated Th17 and CD8 cytotoxic and exhausted effectors, while regulatory and antigen-presenting cells are decreased in comparison with circulation.

### 3.3 Does PCOS alter the normal cytokine and cellular immune signature in the follicular and systemic compartments?

**Figure 3:**
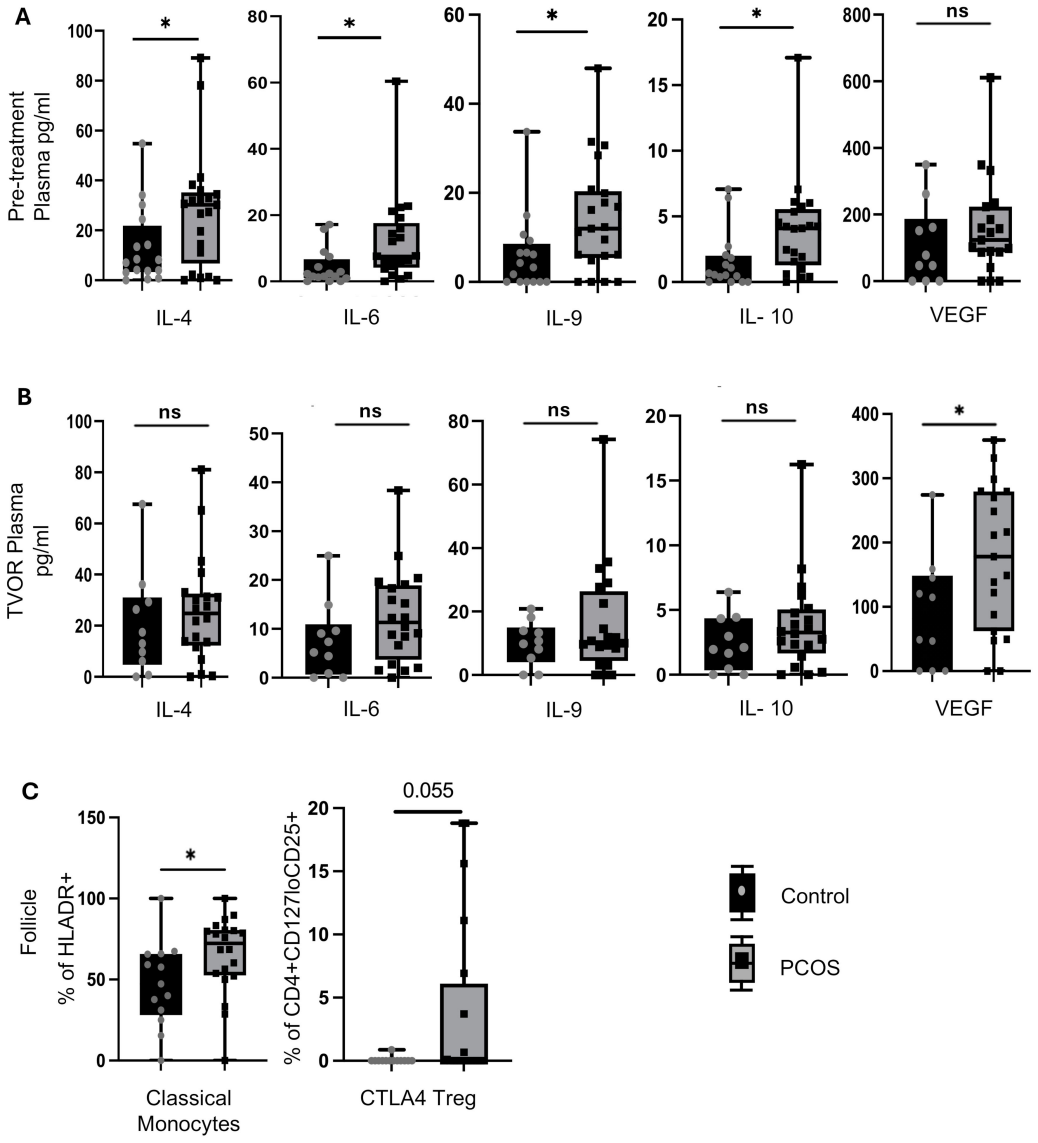
Cytokine and immune cell alterations in PCOS vs control. **(A)** Pre-treatment (Visit 1) plasma in PCOS has higher levels of IL-4, IL-6, IL-9 and IL-10 than control (p<0.05 for all).No significant difference between pre-treatment plasma VEGF in PCOS vs control (p>0.05) **(B)** These differences were not observed at TVOR (visit 2). Plasma levels of IL-4, IL-6, IL-9 and IL-10 were higher in PCOS than control but the difference did not reach level of significance (p>0.05) .However,TVOR plasma has higher VEGF (p<0.05) in PCOS vs control. **(C)** Follicular fluid has higher numbers of classical monocytes (p<0.05) and a trend towards higher CTLA4+ Tregs (p=0.08) in PCOS compared to control. No significant differences in major lymphocyte subsets in blood between PCOS and control at either time point. No significant PCOS vs control differences in other follicular fluid immune subsets. Paired t-tests were used for plasma vs FF comparisons; unpaired t-tests for PCOS vs control (no multiple-test correction). For cytokine analysis n=30 (Control=10, PCOS=20) and for immune cell population study n=33 (Control=13, PCOS=20). Significant differences and trends (0.05<p<0.1) are shown. *p<0.05, n.s, not significant.

Having established the differences in the composition of systemic and follicular compartments, we next investigated the role of PCOS in altering these patterns (**Figure 3**).

Pre-treatment plasma in PCOS has higher levels of IL-4, IL-6, IL-9, and IL-10 than the control (*p* < 0.05) (**Figure 3A**). However, by the time of TVOR, the levels of these cytokines in PCOS were no longer significantly different (**Figure 3B**). With respect to angiogenic growth factors, the pre-treatment plasma VEGF difference was not significant between PCOS and control (**Figure 3A**), but TVOR plasma has higher VEGF (*p* < 0.05) in PCOS (**Figure 3B**). Upon investigating the immune cell composition, we find that PCOS follicular fluid has higher numbers of classical monocytes (*p* < 0.05) and a trend toward higher CTLA4^+^ Tregs compared with the control (0.05 < *p <*0.1) (**Figure 3C**). No significant PCOS vs. control differences were observed in other cell subsets in plasma or FF.

### 3.4 Cellular subset visualization by dimensionality reduction and clustering algorithms

We optimized the use of high-dimensional polychromatic spectral flow cytometry, for the identification of T cells, APCs, and ILCs, simultaneously in the same sample by implementing three 26–28 color panels. In total, we analyzed 33 patient samples (13 control and 20 PCOS) in a series of four experiments as summarized in **Supplementary Table S6**. FlowSom clustering algorithm was combined with tSNE plug-in in FlowJo analysis pipeline to generate tSNE and dimensionality reduction plots that were then superimposed with the FlowSom clustering output to generate the immune landscape of PCOS and control PBMC and follicular immune cells. A heatmap of the median fluorescence intensity (MFI) for every marker analyzed within each cluster was generated to assess phenotypes and assign cell annotation.

**Figure 4:**
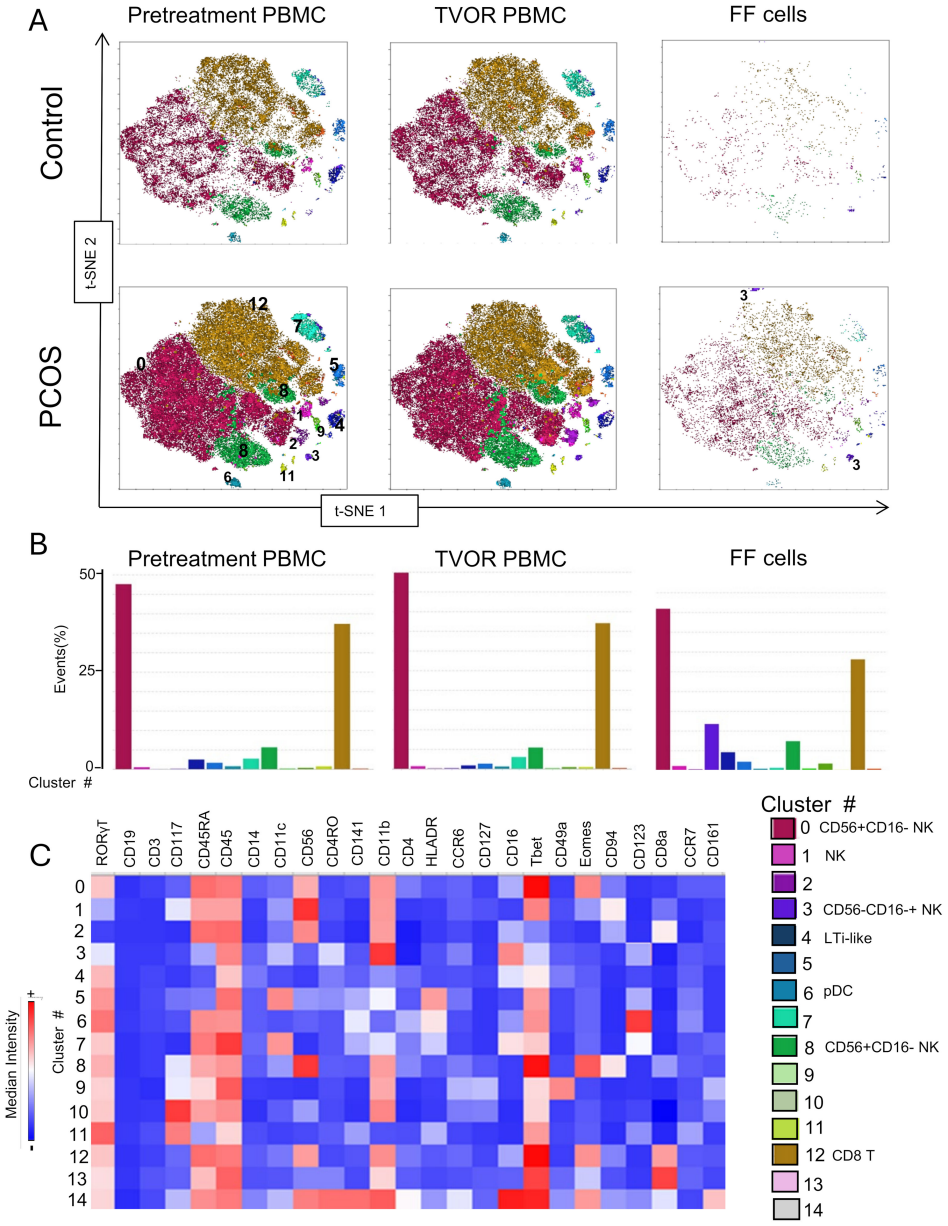
ILC and NK subsets in blood and follicles revealed by tSNE with Flowsom cluster analysis. **(A)** Control and PCOS in pretreatment PBMC, TVOR PBMC, and follicular fluid cells. **(B)** The compositions of clusters in PCOS and control plasma and PBMC are similar with no significant differences. Clusters 0 and 12 are the principal clusters in plasma and follicular fluid. PBMC have a higher proportion of cells in cluster 6, and follicular cells have a higher concentration in cluster 3. The patterns are similar in PCOS and control with no discernable statistical difference in cluster distributions. **(C)** Heatmap of mean fluorescent intensity of each surface and intracellular marker. Experiment with nine samples, three control, and six PCOS.

**Figure 5:**
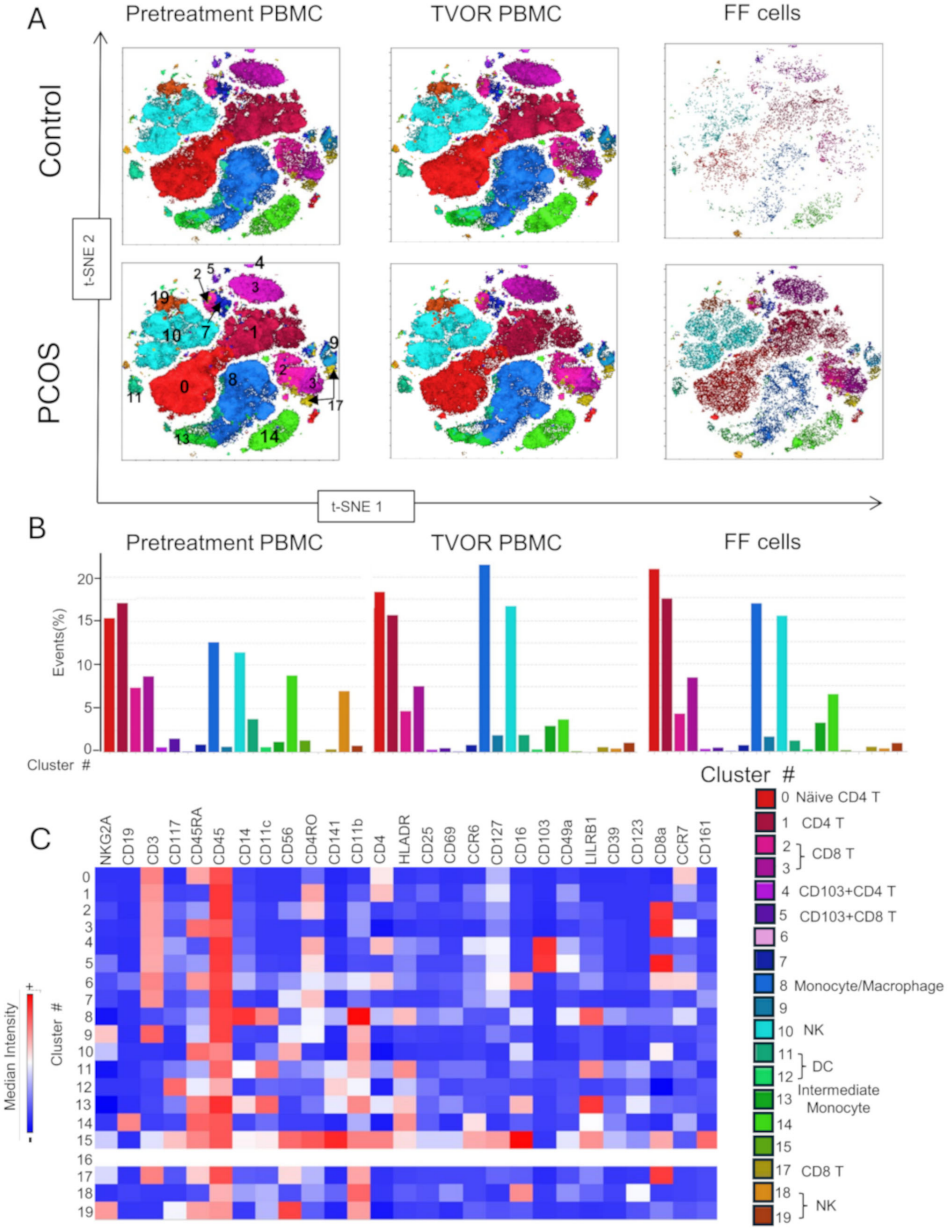
APC and T cell distribution between blood and follicles revealed by tSNE with Flowsom cluster analysis. **(A)** Control and PCOS tSNE plots in pretreatment PBMC, TVOR PBMC, and follicular fluid cells. **(B)** The compositions of clusters in PCOS and control plasma and PBMC are similar with no significant differences. The patterns are similar in PCOS and control with no discernable statistical difference in cluster distributions. **(C)** Heatmap of mean fluorescent intensity of each surface and intracellular marker. Experiment with nine samples, three control, and six PCOS.

**Figure 6:**
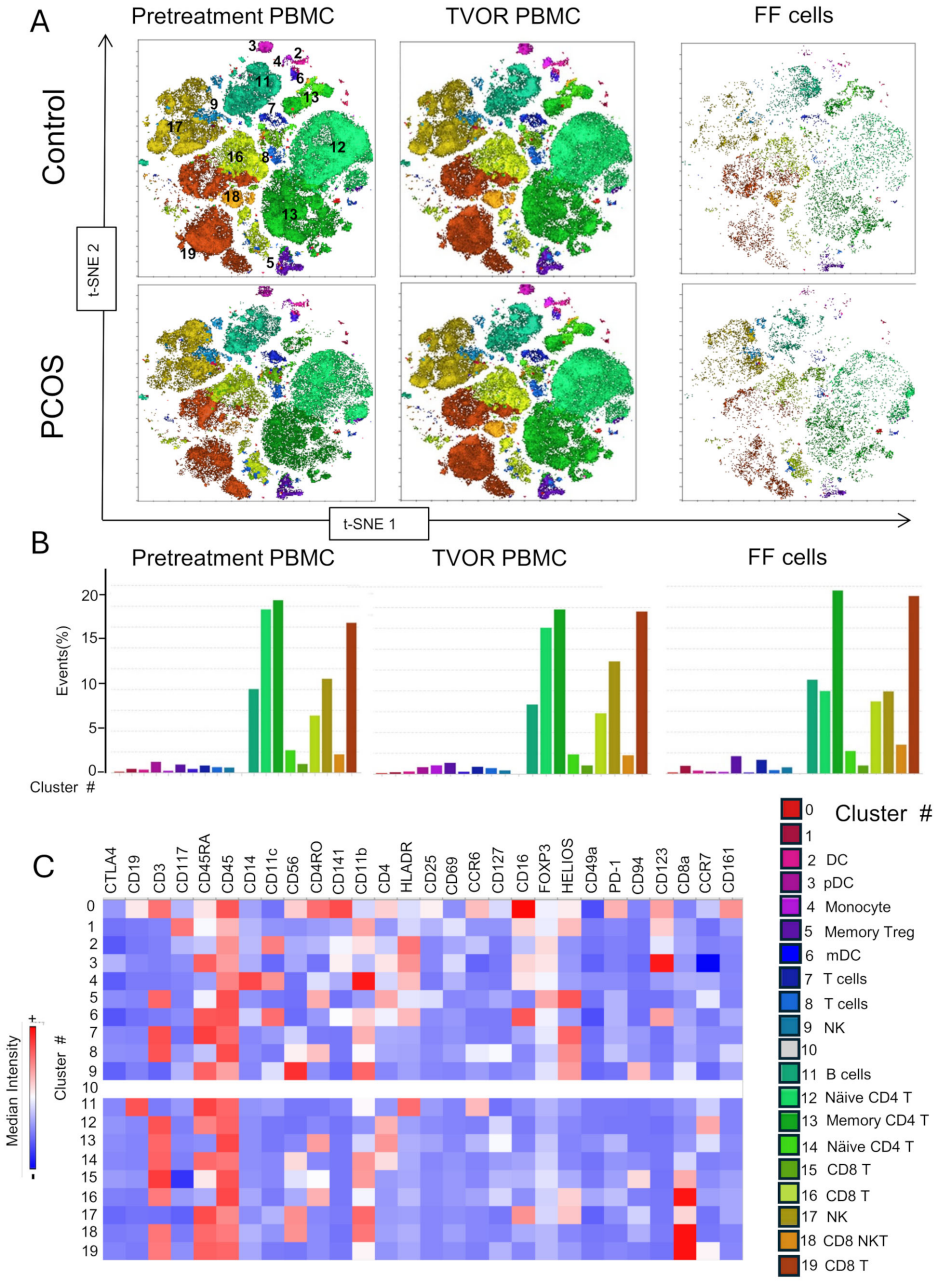
T cell subsets in blood and follicle revealed by tSNE plots with Flowsom cluster analysis. **(A)** Control and PCOS tSNE plots in pretreatment PBMC, TVOR PBMC, and follicular fluid cells. **(B)** The compositions of clusters in PCOS and control plasma and PBMC are similar with no significant differences. The patterns are similar in PCOS and control with no discernable statistical difference in cluster distributions. **(C)** Heatmap of mean fluorescent intensity of each surface and intracellular marker. Experiment with nine samples, three control, and six PCOS.

Dimensional reduction followed by clustering analysis revealed 15 and 20 distinct cellular clusters in ILCs with NK cells and T cell and APC panels, respectively (**Tables 5**–**7**). The ILC panel (**Figure 4**) investigation revealed that cluster 0 (CD56+CD16- NK cells positive for Eomes, RORγt, and Tbet) and cluster 12 (CD 8 T cells) were the most abundant cells in both PBMC and ovarian follicles. The ovarian follicles also had a smaller but distinct population, cluster 3, that was not present in peripheral blood. This population was also comprised of CD56+CD16- NK cells, but they were different from the population identified in the PBMC compartment because they did not express Eomes, RORγt, or Tbet. There was no statistically significant difference by disease status in the immune cluster differentiation between PCOS and controls in any compartment (i.e., *p* < 0.05). The APC panel (**Figure 5**) demonstrated an abundance of monocytes and macrophages in all tissue compartments with no effect of PCOS on the cellular distribution. Similarly, CD4 näive T cells and CD8 T cells were most abundant from the T cell panel (**Figure 6**). The peripheral blood demonstrated a small but distinct plasmacytoid dendritic cell population in both pretreatment and TVOR PBMC (**Figure 4**). A cluster frequency comparison demonstrated that the differences between PCOS vs. control were consistently small and non-significant.

**Table 5:** Cluster analysis of innate lymphoid cell and NK cells. Experiment with nine samples, three control, and six PCOS (**Figure 4**).

Cluster no.	Phenotype	Cell annotation
0	Lin-CD45+CD45RA+CD94-CD56+CD16-CD11b+ CD117-CD127-Tbet+Eomes+RORYT+	CD56+CD16- NK cells
1	Lin-CD45+CD45RA+CD94+CD56+CD16-CD11b+ CD117-CD127-Tbet+	NK cell
2	Lin-CD45+CD45RA+CD94-CD56+CD16-CD11b+ CD117-CD127-RORYT-Tbet-Eomes-CD8a low	NKT cells
3	Lin-CD45+CD94-CD56-CD16+CD11b+CD117-CD127-	CD16+CD56-NK Cells
4	Lin-CD45+CD94-CD56-CD16-CD11b-CD117-CD127-RORYT low	LTi like
5	Lin-CD45+CD45RA+CD94-CD56+CD16-CD11b-CD11c+CD117-CD127-HLADR+RORYT+Tbet+	APC
6	Lin-CD45+CD45RA+CD94-CD56+CD16-HLADR+ RORYT+Tbet+CD123+	pDC
7	Lin-CD45+CD45RA+CD94-CD56-CD16-CD11c+ CD117-CD127-RORYT+Tbet low	Undefined
8	Lin-CD45+CD45RA+CD94+CD56+CD16-CD11b+ CD117-CD127-RORYT-low Tbet+Eomes+	CD56+CD16-NK cells
9	Lin-CD45+CD45RA+CD94-CD56-CD16-CD11b+ CD117-CD127-RORYT+Tbet-Eomes-CD49a+	Undefined
10	Lin-CD45+CD45RA+CD94-CD56-CD16-CD11b+CD117+CD127-RORYT low, Tbet low	LTi like
11	Lin-CD45+CD45RA-CD94-CD56-CD16-CD11b-CD117+CD127-Tbet low RORyT+	LTi like
12	Lin-CD45+CD45RA+CD94-CD56+CD16-CD11b+ CD117-CD127-RORYT +Tbet+Eomes+CD8a+	CD8 T cells
13	Lin-CD45+CD45RA+CD8a+CD94-CD56+CD16-CD11b+CD117-CD127-RORYT+Tbet+Eomes-	CD8 T cell
14	Lin-CD45+CD45RA+CD45RO+CD94-CD56+ CD16+ CD11b+CD141+CD117-CD127+CD161 low RORYT+Tbet+Eomes+	Undefined

The compositions of clusters in PCOS vs. control follicular fluid and PBMC are similar with no discernable statistical difference in cluster distributions.

**Table 6:** Cluster analysis of antigen-presenting cells and T cells. Experiment with nine samples, three control, and six PCOS (**Figure 5**).

Cluster no.	Phenotype	Cell annotation
0	CD45+CD3+CD4+CD45RA+CCR7+	Näive CD4 T cells
1	CD45+CD3+CD4+CD45RO+	CD4 memory T cells
2	CD45+CD3+CD8a+CD45RO+	CD8 memory T cells
3	CD45+CD3+CD8a+CD45RA+	CD8 effector T cells
4	CD45+CD45RO+CD3+CD4+CD103+	CD103+ CD4 T cells
5	CD45+CD3+CD8a+CD45RO+CD103+	CD103+ CD8 T cells
6	CD45+CD45RA+CD3+CD4+CD8a+CD16+CCR7+	Undefined
7	CD45+CD3+CD45RO+	Memory T cells
8	CD45+CD14+CD11c+CD11b+HLADR+LILRB1+	Monocyte/Macrophage
9	CD45+CD3+CD11b+ NKG2A+	Undefined
10	CD45+CD45RA+CD56+CD11b+	NK cell
11	CD45+CD11c+CD11b+HLADR+LILRB1	Monocyte or myeloid dendritic cell
12	CD45+CD117+CD141 low	Dendritic cell
13	CD45+CD45RA+CD14+CD11c+CD11b+HLADR+CD16 low LILRB!+	Intermediate monocyte
14	CD19+CD45+CD45RA+HLADR+CCR6 low	B cell
15	CD45+CD45RA+CD141+CD11c+CD56+CD45RO+CD141+CD11b+CD4+HLADR+CD117 low CCR6+ CD127+ CD16+ CD8a+ LILRB1+	Complex set of surface markers—undefined
16	No features	No features
17	CD45+CD3+CD8a+CD45RA+	Näive CD8 T cells
18	CD45+CD16+CD11b+	NK Cells
19	CD45+CD45RA+CD56+CD11b+NKG2A	NK cell

The compositions of clusters in PCOS vs. control follicular fluid and PBMC are similar with no discernable statistical difference in cluster distributions.

**Table 7:** Cluster analysis of T cell subsets. Experiment with six samples, three control, and three PCOS (**Figure 6**).

Cluster no.	Phenotype	Cell annotation
0	CD45+CD3+CD45RO+CD141+CD16++PD-1, CD123low,CD161+CCR6+	Undefined
1	CD45+CD117+CD11b+CD16+HELIOS+CD123low	Undefined
2	CD45+CD11c+CD11b+HLADR+	DC
3	D45+CD45RA+HLADR+CD16 low CD123+	pDC
4	CD45+CD14+CD11c+CD11b+	Monocyte
5	CD45+CD3+CD4+CD45RO+FOXP3+HELIOS+	Memory Treg
6	CD45+CD45RA+CD11c+CD11b+HLDR+CD16+CD123 low	Dendritic cells (mDC)
7	CD45+CD45RA+CD3+CD11b+HELIOS	T cells
8	CD45+CD45RO+CD3+HELIOS+	T cells
9	CD3-CD45+CD45RA+CD56+CD11b+HELIOS+CD94+	NK cells
10	NO FEATURES	
11	CD45+CD45RA+CD19+CCR6+HLADR+	B cell
12	CD45+CD45Ra+CD3+CD4+CCR7	Näive CD4 T cell
13	CD45+ CD3+CD45RO+CD4	CD4 memory T cell
14	CC45+CD3+CD4+CD45RA+CD11b	CD4 T cell
15	CD45+CD3+CD11b+CD8a+	CD8 T cells
16	CD45+CD3+CD45RO+CD8a+HELIOS low	CD8 T cells
17	CD3-CD45+CD45RA+CD16+CD56+CD11b+HELIOS low CD8a low	NK cell
18	CD3+CD45+CD45RA+CD56 med+CD11b+CD8a+	CD8+NKT cells
19	CD45+CD45RA+CD3+HELIOS+CD8a+	CD8 T cells

The compositions of clusters in PCOS vs. control follicular fluid and PBMC are similar with no discernable statistical difference in cluster distributions.

## 4 Discussion

In this study, we characterized immunological differences between the systemic circulation and the ovarian follicle and investigated how these differences manifest in women with PCOS. Our results underscore several core findings. Systemically, we observed higher IL-2, IL-4, IL-9, IL-17A, TNF-α, and MCP-1 in TVOR plasma compared with follicular fluid, whereas follicular fluid was markedly enriched in angiogenic factors (VEGF and EGF) and displayed distinct immune cellular composition—including reduced plasmacytoid dendritic cells, classical monocytes, Treg subsets, and naïve/central memory CD4 T cells. Conversely, the follicular fluid exhibited higher frequencies of Th17 and PD-1-expressing CD4 T cells and CD69+ cytotoxic CD8 T cells, suggesting a specialized local immune environment. Interestingly and an object of further study, we observed trends toward the enrichment of innate lymphocytes, ILC1s, in follicular fluid. Taken together, the finding of differential sequestration of cytokines and angiogenic factors between the systemic circulation and the ovarian follicle reveals unique immune microenvironments, indicating that the measurement of systemic immune parameters alone does not reflect ovarian activity and necessitating local sampling to understand the pathophysiology in PCOS. In other words, immune processes within the follicle may operate somewhat independently of peripheral blood immune status, reinforcing the need to directly assess the ovarian microenvironment. Immune cells in the follicle (macrophages, T cells, dendritic cells, etc.) play essential roles in folliculogenesis and ovulation (42). Perturbations in this localized immune network, for example, a proinflammatory cytokine milieu or altered immune cell activation in PCOS follicular fluid, could directly impair oocyte development and ovulatory function.

Our findings also clarify how PCOS status shapes local versus systemic immune signatures. Clinically, altered cytokine patterns have been noted during the normal menstrual cycle (43–45) as well as menstrual disorders like dysmenorrhea (46, 47). Pro-inflammatory cytokines (e.g., IL-1β, IL-6, and TNF-α) are elevated especially during the late follicular phase or ovulation and the luteal phase, close to menstruation (43). A study examining peripheral blood monocyte gene expression in primary dysmenorrhea (46) found that pro‐inflammatory cytokine genes (e.g., IL-1β, TNF-α, and IL-6, IL-8) were upregulated during the menstrual phase relative to unaffected controls, while transcripts related to anti‐inflammatory and tissue repair pathways (such as certain TGF-β family members) were downregulated. PCOS may alter the normal cyclical cytokine signature. Several studies have demonstrated elevated proinflammatory cytokine markers in PCOS predominantly during the early follicular phase (11, 13, 18, 19). In our study, PCOS patients showed higher IL-4, IL-6, IL-9, and IL-10 in pre-treatment plasma, which were no longer elevated by the time of TVOR. Controlled ovarian hyperstimulation may be affecting this reduction, potentially masking an aspect of the inherent PCOS-related inflammatory signals by the time of oocyte retrieval due to the administration of exogenous FSH and LH, which correct follicular development. Interestingly, the increase in IL-6 in PCOS was accompanied by an increase in IL-10 (a key anti-inflammatory cytokine), suggesting a complex immune milieu rather than a uniformly “proinflammatory” state in PCOS. It is possible that compensatory anti-inflammatory mechanisms (such as IL-10 secretion by regulatory T cells) are upregulated alongside pro-inflammatory factors in PCOS.

However, higher VEGF was noted in PCOS TVOR plasma, and the follicular compartment in PCOS contained more classical monocytes and a non-significant trend toward increased CTLA4+ Tregs (an immunomodulatory subset). The presence of more “exhausted” or checkpoint-expressing Tregs might represent a compensatory response attempting to counteract inflammation in the PCOS follicular environment. Emerging evidence, in fact, points to a Th17/Treg imbalance in PCOS, with a bias toward pro-inflammatory Th1/Th17 activity and reduced anti-inflammatory Treg function (36, 48)—for instance, peripheral CD4+CD25+FoxP3+ Tregs have been found to be significantly lower in PCOS women than in controls, while Th17 cells and IL-17 levels are elevated (36). Such an imbalance could foster a chronic inflammatory milieu and disrupt immune tolerance in ovarian tissue. In our study, we did not observe significant differences in Th17 or Treg cell frequencies between PCOS and control within the follicle or blood; however, the baseline elevation of IL-17A and the trend toward higher CTLA4+ Tregs in PCOS follicular fluid are consistent with a subtly altered Th17/Treg axis. These data highlight the need for deeper functional analyses of T cell subsets in PCOS to determine whether an intrinsic Th17/Treg dysregulation contributes to follicular dysfunction.

The contrasting patterns of angiogenic factors between plasma and follicles highlight the importance of localized vascular remodeling, which may be vital for adequate follicle maturation and subsequent luteal function. Notably, immune–angiogenic interactions likely play a significant role in PCOS pathophysiology. Chronic inflammation can promote angiogenesis—for example, pro-inflammatory macrophages secrete angiogenic factors that enhance neovascularization. Consistent with this, women with PCOS show increased ovarian stromal blood flow and elevated VEGF levels (49, 50). Our observation of higher plasma angiopoietin 2 in comparison to follicular fluid in the control group may have significant implications. Angiopoietin-1 (Ang-1) is a potent angiogenic growth factor, whereas angiopoietin-2 (Ang-2) is a vascular disruptive agent (51, 52). Ang-1 and Ang-2 play crucial roles in follicular angiogenesis and growth, particularly during the preovulatory period (53). Studies have demonstrated that the Ang-1 levels are significantly lower and the Ang-2 levels are higher in FF compared to serum (53). One study demonstrated consistently elevated serum Ang-1 levels in PCOS patients compared with the controls, while the serum Ang-2 and follicular Ang-1 levels remained unchanged (54). In this context, a higher level of Ang-2 in control plasma is significant as it may serve a critical role in preventing excessive vascularization by selectively destabilizing neovascular beds, allowing for controlled vascular remodeling in normal ovaries. In PCOS, the lack of systemic Ang-2 upregulation might reflect a failure to balance vascular stability vs. remodeling, leading to persistent vasculature, fibrosis, and impaired folliculogenesis. Metabolic signals may further modulate this angiogenic milieu: hyperinsulinemia and elevated IGF-1 in PCOS have been linked to higher VEGF levels and greater ovarian vascularity (55)​. Our data support the idea that there is a bidirectional communication between the endocrine and immune systems. Consequently, a detailed understanding of the immune cell interactions at the ovarian level is critical to disentangling the molecular pathways involved in PCOS pathogenesis.

Finally, the approach used in this study—high-dimensional spectral flow cytometry combined with clustering algorithms (**Figures 4**–**6**)—provided a comprehensive view of immune cell phenotypes across multiple compartments and revealed no distinct statistically significant differences in immune cell subset distribution between PCOS and control in either blood or follicular compartments. However, we detected subtle population differences that indicate that PCOS does not grossly alter the quantities of major immune cell types. The immune contribution to PCOS may instead lie in qualitative changes to cell function (cytokine secretion, activation state, and signaling pathways) rather than major shifts in cell subset numbers. The ILC panel (**Figure 4**) demonstrated a small NK‐cell-like (CD56+CD16-) cluster 3 in the follicular fluid that differs phenotypically from more conventional NK cells in peripheral blood. It lacks the classical NK cell transcription factors, notably T‐bet or Eomes, and does not conform to the typical CD56^bright^/CD56^dim^ classification used in peripheral blood. Because it lacks the strong cytotoxic phenotype of conventional NK cells (CD16+T‐bet+, Eomes+), this cluster could serve more tissue‐modulatory or immunoregulatory roles in the follicle, paralleling how “tissue‐resident” or specialized NK cells can promote local tissue remodeling, angiogenesis, and tolerance in other reproductive tissues (25, 56). Similar populations—albeit most famously characterized in the uterus (decidual NK cells) (56–59)—have been described in the literature as specialized ILC/NK subsets adapted to local tissue demands rather than systemic patrol or cytotoxic killing. Additionally, the peripheral blood demonstrated a small but distinct plasmacytoid dendritic cell population in both pretreatment and TVOR PBMC. Although this is a “negative” result, this finding should inform future studies that simply enumerating immune cell populations (especially in peripheral blood) may be insufficient and that functional assays or tissue-specific analyses are needed to uncover PCOS-related immune dysregulation.

Taken together, these data underscore the importance of considering both local and systemic immune perturbations in PCOS. They point toward the value of integrative immunologic approaches to improve our understanding of PCOS and to develop targeted therapies. PCOS pathophysiology likely involves bidirectional interactions between the immune and endocrine systems. Chronic low-grade inflammation in PCOS has been linked to metabolic factors such as visceral adiposity and insulin resistance, wherein activated immune cells (e.g., M1 macrophages in adipose tissue) secrete cytokines that exacerbate insulin resistance and hormonal imbalance (42). Conversely, endocrine abnormalities (e.g., hyperandrogenemia) can modulate immune profiles (as seen in animal models of PCOS) (60), creating a vicious cycle in which hormonal and immune disturbances reinforce one another. A holistic understanding of these immune–endocrine interactions, especially within tissue microenvironments like the ovarian follicle, will be critical to design better interventions. Future studies may build on our findings by investigating immune cell function (e.g., cytokine production, cytotoxicity, and signaling) and gene expression in PCOS as well as assessing therapeutic strategies (such as anti-inflammatory or immune-modulating treatments) to restore reproductive and metabolic balance in PCOS.

Lastly, we acknowledge that our study has several limitations that should be considered when interpreting the findings. First, the modest sample size, particularly for certain subgroup analyses, may limit the statistical power to detect subtle differences. Although we matched groups by age and BMI, residual heterogeneity in PCOS phenotypes and metabolic profiles could confound interpretations. Second, our study population consisted of women undergoing IVF, which may introduce selection bias and limit the external generalizability to all individuals with PCOS. Third, while controlled ovarian stimulation allowed us to access follicular fluid, it may have influenced immune parameters. We specifically note the normalization of cytokines by the time of TVOR, suggesting that future studies in unstimulated cycles (e.g., trials of *in vitro* maturation protocols) or *in vivo* follicle development in animal models could be informative.

In summary, we report that women with PCOS exhibit distinct immune signatures in the ovarian follicular fluid compared to circulation, with clear compartment-specific patterns that are largely preserved regardless of PCOS status. While systemic cytokine levels hint at an inflammatory state in PCOS, these do not reflect the ovarian immune environment, emphasizing that local ovarian sampling is indispensable for immunological studies of PCOS. We also show that PCOS is associated with only subtle changes in immune cell composition, suggesting that the syndrome’s immune pathology may involve functional dysregulation rather than overt immune cell population shifts. This work provides a foundation and a high-dimensional cellular dataset for future research to dissect the immune–reproductive interface in PCOS. Our findings encourage a shift in focus toward immune cell function and tissue-level immunity in PCOS, which could unveil novel targets to improve fertility and metabolic outcomes in this common disorder.

## Data Availability

The raw data supporting the conclusions of this article will be made available by the authors, without undue reservation.
