# Supplementary material for: High-dimensional immune profiling of follicular fluid and systemic circulation reveals distinct immune signatures in women with polycystic ovary syndrome
Source: Front Immunol. 2025 Aug 11;16:1628031. doi: 10.3389/fimmu.2025.1628031 (PMC12375645; doi:10.3389/fimmu.2025.1628031)
Supplement: Supplementary file 1 [file Table1.docx]

**Supplementary Figure Legends**

**Supplementary Figure S1: Antigen Presenting Cells (APC) cells gating scheme in PBMCs.** **(A)** Gating for CD19, CD3, and CD56 negative CD45+HLADR+ cells. **(B)** Monocytes**:** There are 3 types based on CD14 and CD16 expression. Classical Monocytes (cMO) HLADR+CD14+CD16-, Non-Classical Monocytes (ncMO) HLADR+CD14-CD16+and Intermediate Monocytes (iMO) HLADR+CD14+CD16+. **(C)** Dendritic cells (DC):DC are HLADR+CD14-CD16-. There are two types, Plasmacytoid DC (pDC) HLADR+CD14-CD16-CD11c-CD123+ and Myeloid DC(mDC) HLADR+CD14-CD16-CD123-CD11c+CD141+.

**Supplementary Figure S2: T Lymphocytes gating scheme in Peripheral Blood Mononuclear Cells (PBMCs).** **(A)** Gating CD4 and CD8 Lymphocytes. **(B)** Gating of CD4 T Lymphocytes. CD4 CM (Central Memory) CCR7+CD45RA-, CD4 EM (Effector Memory) CCR7-CD45RA-, CD4 Näive CCR7+CD45RA+, CD4 T eff (T effector) CCR7-CD45RA+, CD4 Exhausted PD-1 +, CD4 Activated CD69+, CD4 Th17 CCR6+CD161+. **(C)** Gating of CD8 T Lymphocytes. CD8 CM (Central Memory) CCR7+CD45RA-, CD8 EM (Effector Memory) CCR7-CD45RA-, CD Näive CCR7+CD45RA+, CD8 T eff (T effector) CCR7-CD45RA+, CD4 Exhausted PD-1+, CD8 Activated CD69+,CD8 TEMRA CCR7-CD45RA+LILRB1+ **(D)** Regulatory T( CD4) Treg cells. CD4+ cells that are CD25+CD127lo and Transcription Factor FOXP3 +. Näive Treg cells CD45RA+ and Memory Tregs CD45RO+. Activated Tregs express HLADR . Other markers for Tregs Helios, CTLA4, CD39.

**Supplementary Figure S3: Innate lymphoid cells gating scheme in Ovarian follicular fluid.** ILCs are Lineage negative cells(Lin-) **(A)** Gating for lineage negative CD45+ cells. Lineage consists of CD3, CD14, and CD19. **(B)NK cells** (CD56+CD94+ CD117-CD127-) NK cells are of two types based on CD56 and CD16 expression level- CD56 bright and CD56dim. **(C) LTi-like** (Lin-CD56-CD94-CD117+CD127+RORyT+) cells. **(D) ILC1** (Lin-CD94-CD56+CD117+CD127+ and transcription factor Tbet+) **(E) ILC3** (Lin-CD94-CD56+CD117+CD127+ and transcription factor RORyT+)

**Supplementary Figures**


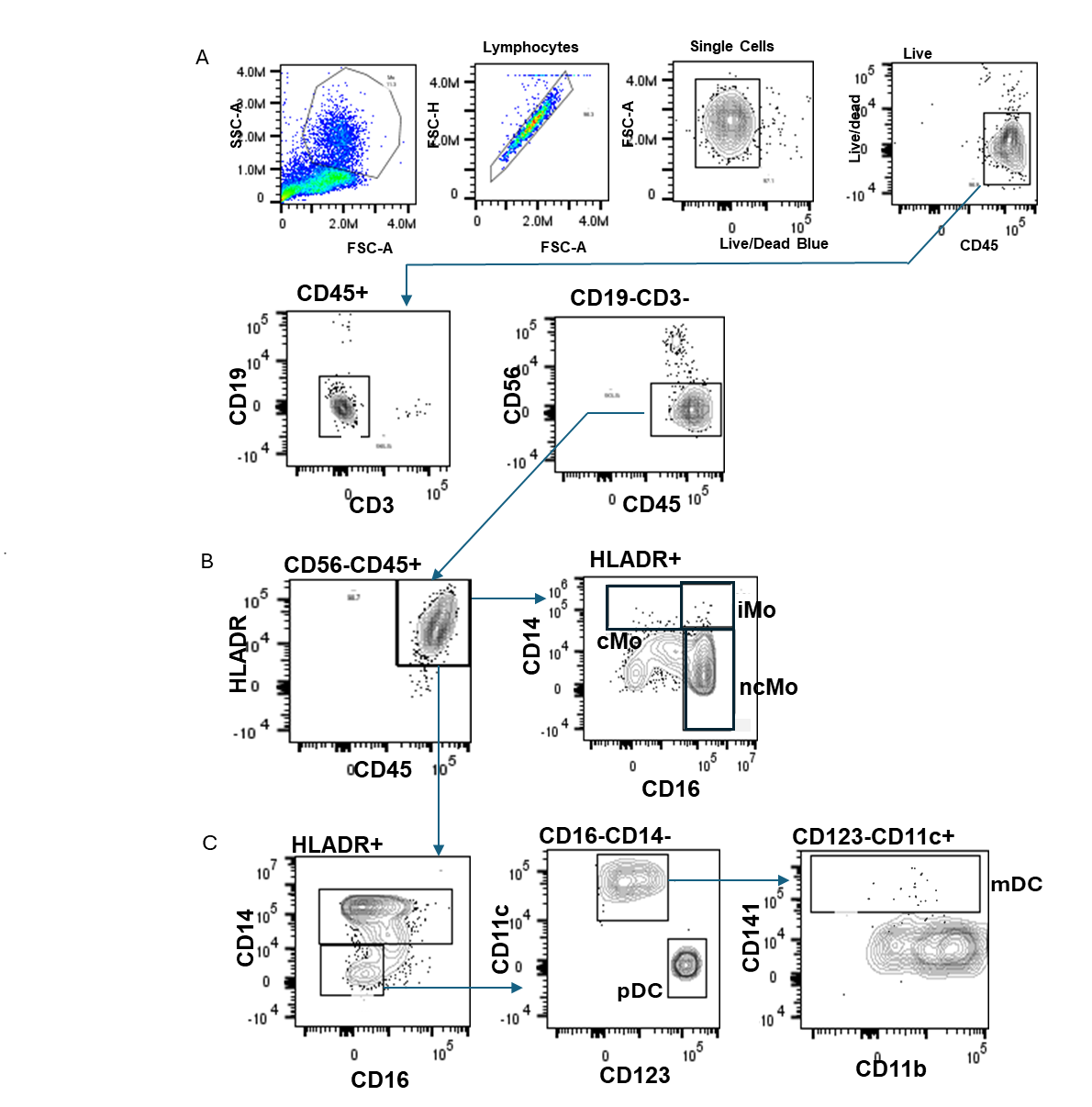
**Figure S1. Antigen Presenting Cells (APC) cells gating scheme in PBMCs. (A)** Gating for CD19, CD3, and CD56 negative CD45+HLADR+ cells. **(B)** Monocytes: There are 3 types based on CD14 and CD16 expression. Classical Monocytes (cMO) HLADR+CD14+CD16-, Non-Classical Monocytes (ncMO) HLADR+CD14-CD16+and Intermediate Monocytes (iMO) HLADR+CD14+CD16+. **(C)** Dendritic cells (DC):DC are HLADR+CD14-CD16-. There are two types, Plasmacytoid DC (pDC) HLADR+CD14-CD16-CD11c-CD123+ and Myeloid DC (mDC) HLADR+CD14-CD16-CD123-CD11c+CD141+.

**
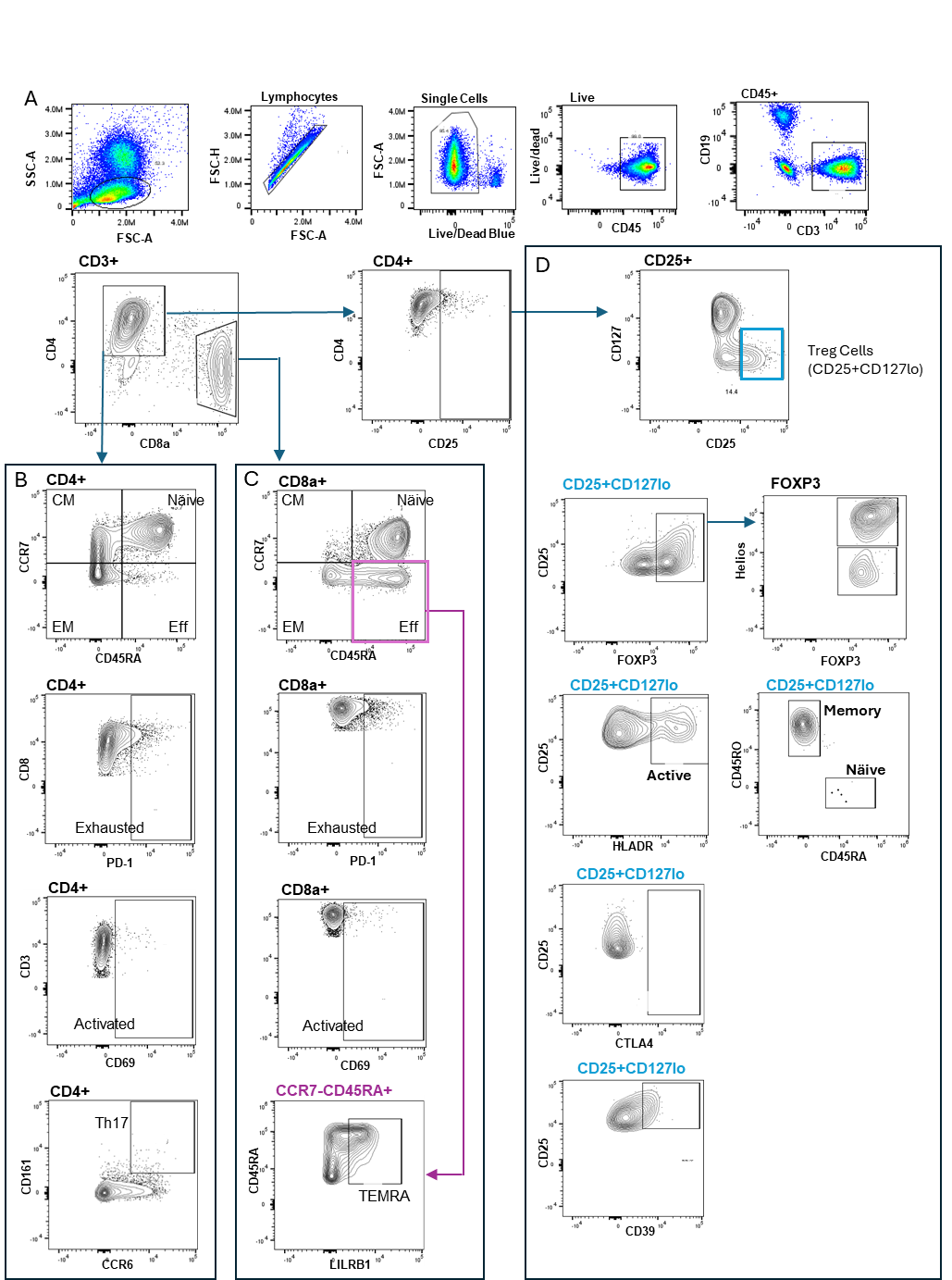
**

**Figure S2. T Lymphocytes gating scheme in Peripheral Blood Mononuclear Cells (PBMCs).** **(A)** Gating CD4 and CD8 Lymphocytes. **(B)** Gating of CD4 T Lymphocytes. CD4 CM (Central Memory) CCR7+CD45RA-, CD4 EM (Effector Memory) CCR7-CD45RA-, CD4 Näive CCR7+CD45RA+, CD4 T eff (T effector) CCR7-CD45RA+, CD4 Exhausted PD-1 +, CD4 Activated CD69+, CD4 Th17 CCR6+CD161+. **(C)** Gating of CD8 T Lymphocytes. CD8 CM (Central Memory) CCR7+CD45RA-, CD8 EM (Effector Memory) CCR7-CD45RA-, CD Näive CCR7+CD45RA+, CD8 T eff (T effector) CCR7-CD45RA+, CD4 Exhausted PD-1+, CD8 Activated CD69+,CD8 TEMRA CCR7-CD45RA+LILRB1+ **(D)** Regulatory T( CD4) Treg cells. CD4+ cells that are CD25+CD127lo and Transcription Factor FOXP3 +. Näive Treg cells CD45RA+ and Memory Tregs CD45RO+. Activated Tregs express HLADR. Other markers for Tregs Helios, CTLA4, CD39.


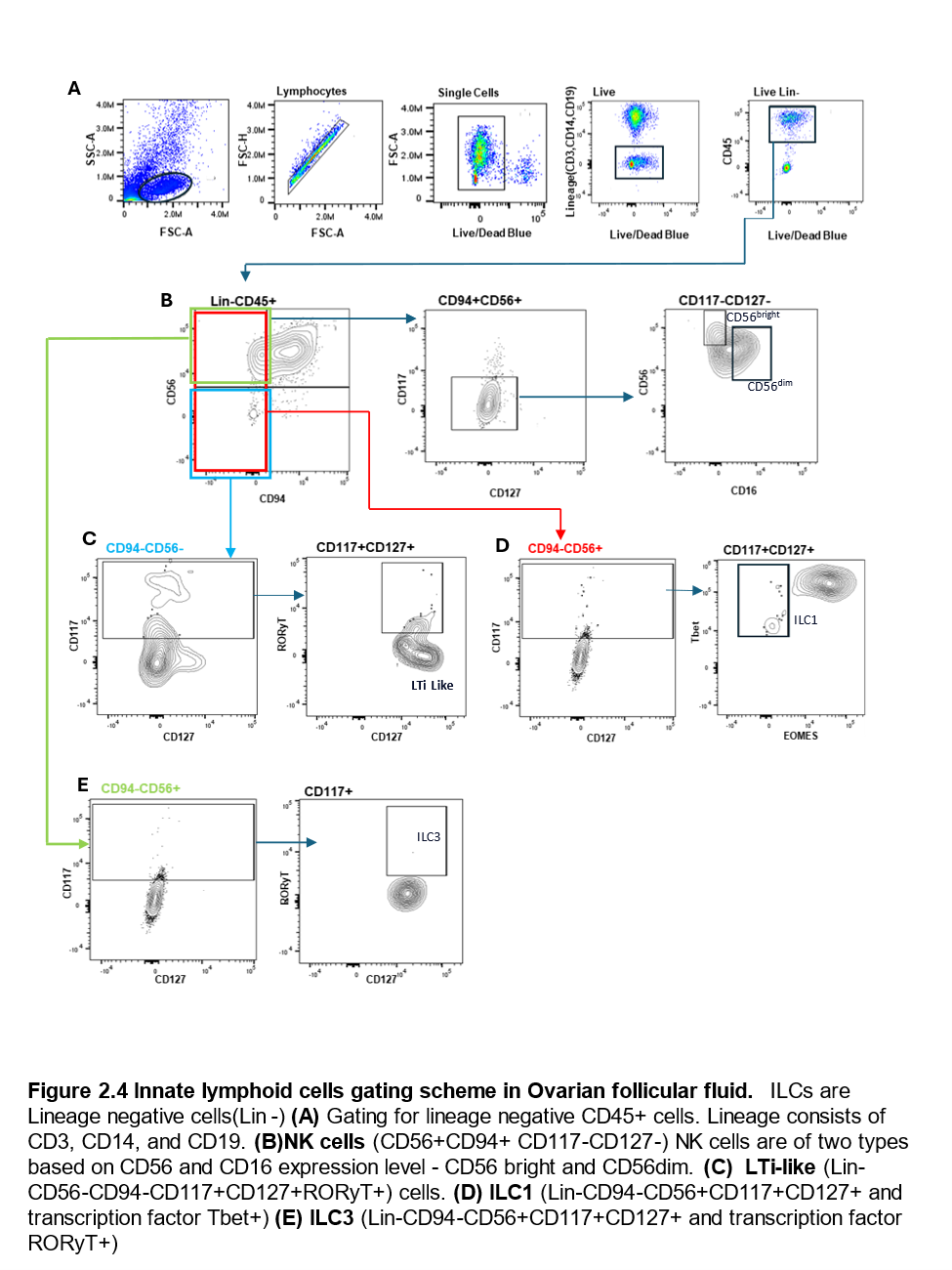


**Figure S3. Innate Lymphoid cells gating scheme in Ovarian follicular fluid**. ILCs are Lineage negative cells (Lin-) **(A)** Gating for Lineage negative CD45+ cells. Lineage consists of CD3, CD14 and CD19. **(B)** NK cells (CD56+CD94+CD117-CD127-). NK cells are of two typws based on CD56 and CD16 expression level-CD56 bright and CD56 dim**. (C)** LTi-like (Lin-CD56-CD94-CD117+CD127+RORyT+) cells. (D) ILC1 (LIN-CD94-CD56+CD117+CD127+ Tbet+) (E) ILC3 (Lin-CD94-CD56+CD117+CD127+RORyT+

**Supplementary Tables**

**Supplementary Table S1: Statistical summary of Cytokine and Angiogenic factors compared between Plasma and Follicular Fluid at TVOR**

| **Analyte** | **Comparison** | **Test** | **p-value** | **Significance** | **Summary** |
| --- | --- | --- | --- | --- | --- |
| IL-2 | TVOR Plasma vs Follicular Fluid | paired student's t-test | **<0.0001** | ******** | **Higher in TVOR Plasma** |
| IL-4 | TVOR Plasma vs Follicular Fluid | paired student's t-test | **<0.0001** | ******** | **Higher in TVOR Plasma** |
| IL-5 | TVOR Plasma vs Follicular Fluid | paired student's t-test | 0.2 | ns | Not significant |
| IL-6 | TVOR Plasma vs Follicular Fluid | paired student's t-test | 0.3 | ns | Not significant |
| IL-9 | TVOR Plasma vs Follicular Fluid | paired student's t-test | **0.0002** | ******* | **Higher in TVOR Plasma** |
| IL-10 | TVOR Plasma vs Follicular Fluid | paired student's t-test | 0.6 | ns | Not significant |
| IL-13 | TVOR Plasma vs Follicular Fluid | paired student's t-test | 0.2 | ns | Not significant |
| IL-17A | TVOR Plasma vs Follicular Fluid | paired student's t-test | **<0.0001** | ******** | **Higher in TVOR Plasma** |
| IL-17F | TVOR Plasma vs Follicular Fluid | paired student's t-test | 0.1 | ns | Not significant |
| IL-21 | TVOR Plasma vs Follicular Fluid | paired student's t-test | 0.3 | ns | Not significant |
| IL-22 | TVOR Plasma vs Follicular Fluid | paired student's t-test | 0.1 | ns | Not significant |
| IL-27 |  |  |  |  | **Undetected in any sample** |
| IFNγ | TVOR Plasma vs Follicular Fluid | paired student's t-test | 0.13 | ns | Not significant |
| TNFα | TVOR Plasma vs Follicular Fluid | paired student's t-test | **0.0025** | ****** | **Higher in TVOR Plasma** |
| IL-1α | TVOR Plasma vs Follicular Fluid | paired student's t-test | 0.15 | ns | Not significant |
| IL-1 β | TVOR Plasma vs Follicular Fluid | paired student's t-test | 0.5 | ns | Not significant |
| IL-11 | TVOR Plasma vs Follicular Fluid | paired student's t-test | 0.15 | ns | Not significant |
| IL-15 | TVOR Plasma vs Follicular Fluid | paired student's t-test | 0.18 | ns | Not significant |
| GM-CSF | TVOR Plasma vs Follicular Fluid | paired student's t-test | 0.06 | ns | Not significant |
| IL-18 | TVOR Plasma vs Follicular Fluid | paired student's t-test | 0.5 | ns | Not significant |
| IL-23 | TVOR Plasma vs Follicular Fluid | paired student's t-test | 0.19 | ns | Not significant |
| IL-33 | TVOR Plasma vs Follicular Fluid | paired student's t-test | 0.33 | ns | Not significant |
| IFNα2 | TVOR Plasma vs Follicular Fluid | paired student's t-test | 0.13 | ns | Not significant |
| IL-12p40 | TVOR Plasma vs Follicular Fluid | paired student's t-test | 0.3 | ns | Not significant |
| IL-12p70 | TVOR Plasma vs Follicular Fluid | paired student's t-test | 0.2 | ns | Not significant |
| TGFβ |  |  |  |  | **Undetected in any sample** |
| Ang-1 | TVOR Plasma vs Follicular Fluid | paired student's t-test | 0.4 | ns | Not significant |
| Ang-2 | TVOR Plasma vs Follicular Fluid in Control group | paired student's t-test | 0.0195 | * | **Higher in TVOR plasma in Control group** |
| MCP-1 | TVOR Plasma vs Follicular Fluid | paired student's t-test | **0.0014** | ****** | **Higher in TVOR Plasma** |
| EGF | TVOR Plasma vs Follicular Fluid | paired student's t-test | **0.0002** | ******* | **Higher in Follicular Fluid** |
| VEGF | TVOR Plasma vs Follicular Fluid | paired student's t-test | **0.0025** | ****** | **Higher in Follicular Fluid** |
| TSLP | TVOR Plasma vs Follicular Fluid | paired student's t-test | 0.6 | ns | Not significant |
| PIGF | TVOR Plasma vs Follicular Fluid | paired student's t-test | 0.8 | ns | Not significant |

**Supplementary Table S2: Statistical summary of Immune cell subsets compared between Peripheral Blood (PBMC) and and Follicular Fluid at TVOR**

| **Cell Type** | **Comparison** | **Test** | **p-value** | **Significance** | **Summary** |
| --- | --- | --- | --- | --- | --- |
| ILC1 | TVOR PBMC vs Follicular Fluid | paired student's t-test | 0.07 | ns | Higher in FF (Trend) |
| LTi | TVOR PBMC vs Follicular Fluid | paired student's t-test | 0.16 | ns | Not significant |
| ILC3 | TVOR PBMC vs Follicular Fluid | paired student's t-test | 0.46 | ns | Not significant |
| CD56 bright NK cells | TVOR PBMC vs Follicular Fluid | paired student's t-test | 0.18 | ns | Not significant |
| CD56dim NK cells | TVOR PBMC vs Follicular Fluid | paired student's t-test | 0.43 | ns | Not significant |
| Plasmacytoid Dendritic Cells (pDC) | TVOR PBMC vs Follicular Fluid | paired student's t-test | **0.007** | ****** | **Higher in PBMC** |
| Myeloid Dendritic Cells (mDC) | TVOR PBMC vs Follicular Fluid | paired student's t-test | 0.23 | ns | Not significant |
| Intermediate Monocyes (iMO) | TVOR PBMC vs Follicular Fluid | paired student's t-test | 0.33 | ns | Not significant |
| Non Classical Monocytes (ncMO) | TVOR PBMC vs Follicular Fluid | paired student's t-test | 0.28 | ns | Not significant |
| Classical Monocytes (cMO) | TVOR PBMC vs Follicular Fluid | paired student's t-test | **0.0086** | ****** | **Higher in PBMC** |
| Th17 | TVOR PBMC vs Follicular Fluid | paired student's t-test | **0.0085** | ****** | **Higher in FF** |
| CD4 T Effector (eff) | TVOR PBMC vs Follicular Fluid | paired student's t-test | 0.44 | ns | Not significant |
| CD4 T Näive | TVOR PBMC vs Follicular Fluid | paired student's t-test | **0.042** | ***** | **Higher in PBMC** |
| CD4 T Central Memory (CM) | TVOR PBMC vs Follicular Fluid | paired student's t-test | **0.025** | ***** | **Higher in PBMC** |
| CD4 T Effector Memory (EM) | TVOR PBMC vs Follicular Fluid | paired student's t-test | 0.274 | ns | Not significant |
| CD4 Exhausted | TVOR PBMC vs Follicular Fluid | paired student's t-test | **0.04** | ***** | **Higher in FF** |
| CD8 T Effector (eff) | TVOR PBMC vs Follicular Fluid | paired student's t-test | 0.49 | ns | Not significant |
| CD8 Näive | TVOR PBMC vs Follicular Fluid | paired student's t-test | 0.51 | ns | Not significant |
| CD8 T Central Memory (CM) | TVOR PBMC vs Follicular Fluid | paired student's t-test | 0.51 | ns | Not significant |
| CD8 T Effector Memory (EM) | TVOR PBMC vs Follicular Fluid | paired student's t-test | 0.36 | ns | Not significant |
| CD8 T Effector Memory RA (TEMRA) | TVOR PBMC vs Follicular Fluid | paired student's t-test | 0.53 | ns | Not significant |
| CD8 active | TVOR PBMC vs Follicular Fluid | paired student's t-test | **0.026** | ***** | **Higher in FF** |
| CD8 Exhausted | TVOR PBMC vs Follicular Fluid | paired student's t-test | 0.116 | ns | Not significant |
| Näive Treg cells | TVOR PBMC vs Follicular Fluid | paired student's t-test | 0.12 | ns | Not significant |
| Memory Tregs | TVOR PBMC vs Follicular Fluid | paired student's t-test | **0.0007** | ******* | **Higher in FF** |
| Activated Tregs | TVOR PBMC vs Follicular Fluid | paired student's t-test | **0.0059** | ****** | **Higher in FF** |
| CTLA4 Treg | TVOR PBMC vs Follicular Fluid | paired student's t-test | 0.079 | ns | Higher in FF(Trend) |

**Supplementary Table S3: Statistical summary of Cytokine and Angiogenic factors compared between Control and PCOS in Pretreatment and TVOR plasma and Follicular Fluid .**

| **Analyte** | **Control Vs PCOS** | **Test** | **p-value** | **Significance** | **Summary** |
| --- | --- | --- | --- | --- | --- |
| IL-2 | Pretreatment Plasma | Unpaired t test | 0.9 | ns | Not significant |
|  | TVOR Plasma |  | 0.5 | ns | Not significant |
|  | Follicular Fluid |  | 0.8 | ns | Not significant |
| IL-4 | Pretreatment Plasma | Unpaired t test | **0.03** | ***** | **Higher in PCOS pretreatment plasma** |
|  | TVOR Plasma |  | 0.5 | ns | Not significant |
|  | Follicular Fluid |  | 0.7 | ns | Not significant |
| IL-5 | Pretreatment Plasma | Unpaired t test | 0.44 | n | Not significant |
|  | TVOR Plasma |  | 0.69 | ns | Not significant |
|  | Follicular Fluid |  | 0.34 | ns | Not significant |
| IL-6 | Pretreatment Plasma | Unpaired t test | **0.01** | ***** | **Higher in PCOS pretreatment plasma** |
|  | TVOR Plasma |  | 0.15 | ns | Not significant |
|  | Follicular Fluid |  | 0.2 | ns | Not significant |
| IL-9 | Pretreatment Plasma | Unpaired t test | **0.02** | ***** | **Higher in PCOS pretreatment plasma** |
|  | TVOR Plasma |  | 0.17 | ns | Not significant |
|  | Follicular Fluid |  | 0.2 | ns | Not significant |
| IL-10 | Pretreatment Plasma | Unpaired t test | **0.02** | ***** | **Higher in PCOS pretreatment plasma** |
|  | TVOR Plasma |  | 0.36 | ns | Not significant |
|  | Follicular Fluid |  | 0.18 | ns | Not significant |
| IL-13 | Pretreatment Plasma | Unpaired t test | 0.06 | ns | Not significant |
|  | TVOR Plasma |  | 0.8 | ns | Not significant |
|  | Follicular Fluid |  | 0.36 | ns | Not significant |
| IL-17A | Pretreatment Plasma | Unpaired t test | 0.15 | ns | Not significant |
|  | TVOR Plasma |  | 0.82 | ns | Not significant |
|  | Follicular Fluid |  | 0.69 | ns | Not significant |
| IL-17F | Pretreatment Plasma | Unpaired t test | 0.15 | ns | Not significant |
|  | TVOR Plasma |  | 0.8 | ns | Not significant |
|  | Follicular Fluid |  | 0.6 | ns | Not significant |
| IL-21 | Pretreatment Plasma | Unpaired t test | 0.18 | ns | Not significant |
|  | TVOR Plasma |  | 0.17 | ns | Not significant |
|  | Follicular Fluid |  | 0.55 | ns | Not significant |
| IL-22 | Pretreatment Plasma | Unpaired t test | 0.22 | ns | Not significant |
|  | TVOR Plasma |  | 0.89 | ns | Not significant |
|  | Follicular Fluid |  | 0.82 | ns | Not significant |
| IFNγ | Pretreatment Plasma | Unpaired t test | 0.12 | ns | Not significant |
|  | TVOR Plasma |  | 0.17 | ns | Not significant |
|  | Follicular Fluid |  | 0.22 | ns | Not significant |
| TNFα | Pretreatment Plasma | Unpaired t test | 0.09 | ns | Not significant |
|  | TVOR Plasma |  | 0.83 | ns | Not significant |
|  | Follicular Fluid |  | 0.76 | ns | Not significant |
| IL-1α | Pretreatment Plasma | Unpaired t test | 0.48 | ns | Not significant |
|  | TVOR Plasma |  | 0.16 | ns | Not significant |
|  | Follicular Fluid |  | 0.2 | ns | Not significant |
| IL-1 β | Pretreatment Plasma | Unpaired t test | 0.36 | ns | Not significant |
|  | TVOR Plasma |  | 0.36 | ns | Not significant |
|  | Follicular Fluid |  | 0.43 | ns | Not significant |
| IL-11 | Pretreatment Plasma | Unpaired t test | 0.43 | ns | Not significant |
|  | TVOR Plasma |  | 0.7 | ns | Not significant |
|  | Follicular Fluid |  | 0.4 | ns | Not significant |
| IL-15 | Pretreatment Plasma | Unpaired t test | 0.3 | ns | Not significant |
|  | TVOR Plasma |  | 0.25 | ns | Not significant |
|  | Follicular Fluid |  | 0.5 | ns | Not significant |
| GM-CSF | Pretreatment Plasma | Unpaired t test | 0.26 | ns | Not significant |
|  | TVOR Plasma |  | 0.18 | ns | Not significant |
|  | Follicular Fluid |  | 0.22 | ns | Not significant |
| IL-18 | Pretreatment Plasma | Unpaired t test | 0.5 | ns | Not significant |
|  | TVOR Plasma |  | 0.9 | ns | Not significant |
|  | Follicular Fluid |  | 0.06 | ns | Not significant |
| IL-23 | Pretreatment Plasma | Unpaired t test | 0.46 | ns | Not significant |
|  | TVOR Plasma |  | 0.5 | ns | Not significant |
|  | Follicular Fluid |  | 0.47 | ns | Not significant |
| IL-33 | Pretreatment Plasma | Unpaired t test | 0.74 | ns | Not significant |
|  | TVOR Plasma |  | 0.5 | ns | Not significant |
|  | Follicular Fluid |  | 0.5 | ns | Not significant |
| IFNα2 | Pretreatment Plasma | Unpaired t test | 0.43 | ns | Not significant |
|  | TVOR Plasma |  | 0.36 | ns | Not significant |
|  | Follicular Fluid |  | 0.2 | ns | Not significant |
| IL-12p40 | Pretreatment Plasma | Unpaired t test | 0.37 | ns | Not significant |
|  | TVOR Plasma |  | 0.5 | ns | Not significant |
|  | Follicular Fluid |  | 0.85 | ns | Not significant |
| IL-12p70 | Pretreatment Plasma | Unpaired t test | 0.67 | ns | Not significant |
|  | TVOR Plasma |  | 0.69 | ns | Not significant |
|  | Follicular Fluid |  | 0.54 | ns | Not significant |
| Angiopoietin 1 | Pretreatment Plasma | Unpaired t test |  |  | **Undetected in pretreatment plasma** |
|  | TVOR Plasma |  | 0.11 | ns | Not significant |
|  | Follicular Fluid |  | 0.33 | ns | Not significant |
| Angiopoietin 2 | Pretreatment Plasma | Unpaired t test | 0.49 | ns | Not significant |
|  | TVOR Plasma |  | 0.96 | ns | Not significant |
|  | Follicular Fluid |  | 0.37 | ns | Not significant |
| EGF | Pretreatment Plasma | Unpaired t test | 0.4 | ns | Not significant |
|  | TVOR Plasma |  | 0.18 | ns | Not significant |
|  | Follicular Fluid |  | 0.14 | ns | Not significant |
| PIGF | Pretreatment Plasma | Unpaired t test |  |  | **Undetected in pretreatment plasma** |
|  | TVOR Plasma |  | 0.14 | ns | Not significant |
|  | Follicular Fluid |  | 0.18 | ns | Not significant |
| VEGF | Pretreatment Plasma | Unpaired t test | 0.33 | ns | Not significant |
|  | TVOR Plasma |  | **0.038** | ***** | **TVOR Plasma in PCOS has higher levels** |
|  | Follicular Fluid |  | 0.16 | ns | Not significant |
| MCP-1 | Pretreatment Plasma | Unpaired t test | 0.69 | ns | Not significant |
|  | TVOR Plasma |  | 0.65 | ns | Not significant |
|  | Follicular Fluid |  | 0.28 | ns | Not significant |
| TSLP | Pretreatment Plasma | Unpaired t test | 0.33 | ns | Not significant |
|  | TVOR Plasma |  | 0.4 | ns | Not significant |
|  | Follicular Fluid |  | 0.5 | ns | Not significant |
| TGFβ | Pretreatment Plasma |  |  |  | **Undetected in any sample** |
|  | TVOR Plasma |  |  |  |  |
|  | Follicular Fluid |  |  |  |  |
| Il-27 | Pretreatment Plasma |  |  |  | **Undetected in any sample** |
|  | TVOR Plasma |  |  |  |  |
|  | Follicular Fluid |  |  |  |  |

**Supplementary Table S4: Statistical summary of Immune Cell subsets compared between Control and PCOS in Pretreatment and TVOR plasma and Follicular Fluid.**

| **Cell Type** | **Control Vs PCOS** | **Test** | **p-value** | **Significance** | **Summary** |
| --- | --- | --- | --- | --- | --- |
| ILC1 | Pretreatment PBMC | Unpaired t test | 0.35 | ns | Not significant |
|  | TVOR PBMC |  | 0.44 | ns | Not significant |
|  | Follicular Fluid |  | 0.65 | ns | Not significant |
| LTi | Pretreatment PBMC | Unpaired t test | 0.14 | ns | Not significant |
|  | TVOR PBMC |  | 0.33 | ns | Not significant |
|  | Follicular Fluid |  | 0.97 | ns | Not significant |
| ILC3 | Pretreatment PBMC | Unpaired t test | 0.2 | ns | Not significant |
|  | TVOR PBMC |  | 0.46 | ns | Not significant |
|  | Follicular Fluid |  | 0.44 | ns | Not significant |
| CD56 bright NK cells | Pretreatment PBMC | Unpaired t test | 0.84 | ns | Not significant |
|  | TVOR PBMC |  | 0.72 | ns | Not significant |
|  | Follicular Fluid |  | 0.27 | ns | Not significant |
| CD56dim NK cells | Pretreatment PBMC | Unpaired t test | 0.91 | ns | Not significant |
|  | TVOR PBMC |  | 0.53 | ns | Not significant |
|  | Follicular Fluid |  | 0.24 | ns | Not significant |
| Plasmacytoid Dendritic Cells (pDC) | Pretreatment PBMC | Unpaired t test | 0.06 | ns | Not significant |
|  | TVOR PBMC |  | 0.057 | ns | Not significant |
|  | Follicular Fluid |  | 0.92 | ns | Not significant |
| Myeloid Dendritic Cells (mDC) | Pretreatment PBMC | Unpaired t test | 0.63 | ns | Not significant |
|  | TVOR PBMC |  | 0.49 | ns | Not significant |
|  | Follicular Fluid |  | 0.3 | ns | Not significant |
| Intermediate Monocyes (iMO) | Pretreatment PBMC | Unpaired t test | 0.42 | ns | Not significant |
|  | TVOR PBMC |  | 0.28 | ns | Not significant |
|  | Follicular Fluid |  | 0.59 | ns | Not significant |
| Non Classical Monocytes (ncMO) | Pretreatment PBMC | Unpaired t test | 0.87 | ns | Not significant |
|  | TVOR PBMC |  | 0.66 | ns | Not significant |
|  | Follicular Fluid |  | 0.43 | ns | Not significant |
| Classical Monocytes (cMO) | Pretreatment PBMC | Unpaired t test | 0.76 | ns | Not significant |
|  | TVOR PBMC |  | 0.44 | ns | Not significant |
|  | Follicular Fluid |  | **0.049** | ***** | **Higher in FC in PCOS** |
| Th17 | Pretreatment PBMC | Unpaired t test | 0.56 | ns | Not significant |
|  | TVOR PBMC |  | 0.82 | ns | Not significant |
|  | Follicular Fluid |  | 0.16 | ns | Not significant |
| CD4 T Effector (eff) | Pretreatment PBMC | Unpaired t test | 0.44 | ns | Not significant |
|  | TVOR PBMC |  | 0.74 | ns | Not significant |
|  | Follicular Fluid |  | 0.23 | ns | Not significant |
| CD4 Näive | Pretreatment PBMC | Unpaired t test | 0.44 | ns | Not significant |
|  | TVOR PBMC |  | 0.74 | ns | Not significant |
|  | Follicular Fluid |  | 0.23 | ns | Not significant |
| CD4 T Central Memory (CM) | Pretreatment PBMC | Unpaired t test | 0.12 | ns | Not significant |
|  | TVOR PBMC |  | 0.93 | ns | Not significant |
|  | Follicular Fluid |  | 0.93 | ns | Not significant |
| CD4 T Effector Memory (EM) | Pretreatment PBMC | Unpaired t test | 0.77 | ns | Not significant |
|  | TVOR PBMC |  | 0.74 | ns | Not significant |
|  | Follicular Fluid |  | 0.57 | ns | Not significant |
| CD4 Exhausted | Pretreatment PBMC | Unpaired t test | 0.88 | ns | Not significant |
|  | TVOR PBMC |  | 0.11 | ns | Not significant |
|  | Follicular Fluid |  | 0.9 | ns | Not significant |
| CD8 T Effector (eff) | Pretreatment PBMC | Unpaired t test | 0.88 | ns | Not significant |
|  | TVOR PBMC |  | 0.93 | ns | Not significant |
|  | Follicular Fluid |  | 0.38 | ns | Not significant |
| CD8 Näive | Pretreatment PBMC | Unpaired t test | 0.72 | ns | Not significant |
|  | TVOR PBMC |  | 0.53 | ns | Not significant |
|  | Follicular Fluid |  | 0.54 | ns | Not significant |
| CD8 T Central Memory (CM) | Pretreatment PBMC | Unpaired t test | 0.07 | ns | Not significant |
|  | TVOR PBMC |  | 0.53 | ns | Not significant |
|  | Follicular Fluid |  | 0.97 | ns | Not significant |
| CD8 T Effector Memory (EM) | Pretreatment PBMC | Unpaired t test | 0.98 | ns | Not significant |
|  | TVOR PBMC |  | 0.38 | ns | Not significant |
|  | Follicular Fluid |  | 0.94 | ns | Not significant |
| CD8 T Effector Memory RA (TEMRA) | Pretreatment PBMC | Unpaired t test | 0.7 | ns | Not significant |
|  | TVOR PBMC |  | 0.81 | ns | Not significant |
|  | Follicular Fluid |  | 0.67 | ns | Not significant |
| CD8 Active | Pretreatment PBMC | Unpaired t test | 0.18 | ns | Not significant |
|  | TVOR PBMC |  | 0.93 | ns | Not significant |
|  | Follicular Fluid |  | 0.22 | ns | Not significant |
| CD8 Exhausted | Pretreatment PBMC | Unpaired t test | 0.63 | ns | Not significant |
|  | TVOR PBMC |  | 0.19 | ns | Not significant |
|  | Follicular Fluid |  | 0.17 | ns | Not significant |
| Näive Treg cells | Pretreatment PBMC | Unpaired t test | 0.72 | ns | Not significant |
|  | TVOR PBMC |  | 0.2 | ns | Not significant |
|  | Follicular Fluid |  | 0.56 | ns | Not significant |
| Memory Tregs | Pretreatment PBMC | Unpaired t test | 0.47 | ns | Not significant |
|  | TVOR PBMC |  | 0.67 | ns | Not significant |
|  | Follicular Fluid |  | 0.37 | ns | Not significant |
| Activated Tregs | Pretreatment PBMC | Unpaired t test | 0.84 | ns | Not significant |
|  | TVOR PBMC |  | 0.32 | ns | Not significant |
|  | Follicular Fluid |  | 0.55 | ns | Not significant |
| CTLA4 Treg | Pretreatment PBMC | Unpaired t test | 0.2 | ns | Not significant |
|  | TVOR PBMC |  | 0.54 | ns | Not significant |
|  | Follicular Fluid |  | **0.055** | **ns** | **Trend of Higher numbers in FC of PCOS** |

**Supplementary Table S5. Statistical summary of Mixed-effects analysis of Compartment, Condition and Condition*Compartment in Cytokines and Immune Cell subsets.**

| **Analyte or Cell Type** | **Fixed Tests** | **F value** | **p-Value** | **Summary of Fixed test result** | **Post‑hoc Tukey *adj-p* (only for factors with p < 0.05)** |
| --- | --- | --- | --- | --- | --- |
| IL-2 | Condition (Control vs PCOS) | 0.30 | 0.58 | Effect of compartment was significant | FF vs TVOR Plasma  **0.01*** |
|  | Compartment (PreTx / TVOR / Follicular) | 10.98 | **<0.001***** |  |  |
|  | Condition × Compartment | 0.41 | 0.66 |  |  |
| IL-4 | Condition (Control vs PCOS) | 0.90 | 0.35 | Effect of compartment was significant | FF vs TVOR Plasma  **0.0001***** |
|  | Compartment (PreTx / TVOR / Follicular) | 13 | **<0.001***** |  |  |
|  | Condition × Compartment | 1.27 | 0.28 |  |  |
| IL-6 | Condition (Control vs PCOS) | 3.04 | 0.09 | Neither disease status, compartment, nor their interaction significant |  |
|  | Compartment (PreTx / TVOR / Follicular) | 0.32 | 0.74 |  |  |
|  | Condition × Compartment | 0.51 | 0.60 |  |  |
| IL-9 | Condition (Control vs PCOS) | 1.61 | 0.21 | Effect of compartment was significant | FF vs TVOR Plasma  **0.0001***** |
|  | Compartment (PreTx / TVOR / Follicular) | 12.9 | **<0.001***** |  |  |
|  | Condition × Compartment | 0.73 | 0.48 |  |  |
| IL-10 | Condition (Control vs PCOS) | 1.8 | 0.18 | Neither disease status, compartment, nor their interaction significant |  |
|  | Compartment (PreTx / TVOR / Follicular) | 0.35 | 0.70 |  |  |
|  | Condition × Compartment | 0.8 | 0.40 |  |  |
| IL-13 | Condition (Control vs PCOS) | 0.98 | 0.32 | Effect of compartment was significant | FF vs TVOR Plasma  **0.03*** |
|  | Compartment (PreTx / TVOR / Follicular) | 4.18 | **0.02*** |  |  |
|  | Condition × Compartment | 0.86 | 0.42 |  |  |
| IL-17A | Condition (Control vs PCOS) | 0.20 | 0.65 | Effect of compartment was significant | FF vs TVOR Plasma  **0.0001***** |
|  | Compartment (PreTx / TVOR / Follicular) | 20.76 | **<0.001***** |  |  |
|  | Condition × Compartment | 0.20 | 0.81 |  |  |
| IFNγ | Condition (Control vs PCOS) | 1.13 | 0.30 | Neither disease status, compartment, nor their interaction significant |  |
|  | Compartment (PreTx / TVOR / Follicular) | 1.42 | 0.24 |  |  |
|  | Condition × Compartment | 0.48 | 0.62 |  |  |
| TNFα | Condition (Control vs PCOS) | 0.30 | 0.58 | Effect of compartment was significant | FF vs TVOR Plasma  **0.0005**** |
|  | Compartment (PreTx / TVOR / Follicular) | 8.89 | **0.0004**** |  |  |
|  | Condition × Compartment | 0.67 | 0.51 |  |  |
| IL-1α | Condition (Control vs PCOS) | 2.59 | 0.11 | Neither disease status, compartment, nor their interaction significant |  |
|  | Compartment (PreTx / TVOR / Follicular) | 1.98 | 0.14 |  |  |
|  | Condition × Compartment | 1.47 | 0.23 |  |  |
| IL-1 β | Condition (Control vs PCOS) | 1.74 | 0.19 | Neither disease status, compartment, nor their interaction significant |  |
|  | Compartment (PreTx / TVOR / Follicular) | 3.07 | 0.054 |  |  |
|  | Condition × Compartment | 1.91 | 0.15 |  |  |
| IL-18 | Condition (Control vs PCOS) | 0.16 | 0.6 | Neither disease status, compartment, nor their interaction significant |  |
|  | Compartment (PreTx / TVOR / Follicular) | 1.73 | 0.18 |  |  |
|  | Condition × Compartment | 4.74 | 0.052 |  |  |
| IL-12p40 | Condition (Control vs PCOS) | 0.31 | 0.58 | Neither disease status, compartment, nor their interaction significant |  |
|  | Compartment (PreTx / TVOR / Follicular) | 0.46 | 0.63 |  |  |
|  | Condition × Compartment | 0.66 | 0.5 |  |  |
| IL-12p70 | Condition (Control vs PCOS) | 0.24 | 0.62 | Overall effect of compartment was significant | Post hoc pairwise comparisons do not reach significance, though differences between FT and Plasma compartment are close (p ≈ 0.07). |
|  | Compartment (PreTx / TVOR / Follicular) | 3.38 | **0.041*** |  |  |
|  | Condition × Compartment | 0.02 | 0.97 |  |  |
| Ang-1 | Condition (Control vs PCOS) | 1.97 | 0.17 | Neither disease status, compartment, nor their interaction significant |  |
|  | Compartment (PreTx / TVOR / Follicular) | 2.2 | 0.11 |  |  |
|  | Condition × Compartment | 0.49 | 0.61 |  |  |
| Ang-2 | Condition (Control vs PCOS) | 0.59 | 0.80 | Significant Compartment × Condition interaction | Significant pairwise difference within Control group only: TVOR Plasma vs. Follicular Fluid.  **p=0.01*** |
|  | Compartment (PreTx / TVOR / Follicular) | 4.07 | **0.04*** |  |  |
|  | Condition × Compartment | 9.07 | **0.001**** |  |  |
| MCP-1 | Condition (Control vs PCOS) | 0.23 | 0.62 | Effect of compartment was significant | FF vs TVOR Plasma  **0.002**** |
|  | Compartment (PreTx / TVOR / Follicular) | 5.38 | **0.007**** |  |  |
|  | Condition × Compartment | 1.17 | 0.316 |  |  |
| EGF | Condition (Control vs PCOS) | 1.52 | 0.227 | Effect of compartment was significant | FF vs TVOR Plasma  **0.0002***** |
|  | Compartment (PreTx / TVOR / Follicular) | 7.93 | **0.0009***** |  |  |
|  | Condition × Compartment | 0.61 | 0.54 |  |  |
| VEGF | Condition (Control vs PCOS) | 3.15 | 0.08 | Effect of compartment was significant | FF vs TVOR Plasma  **0.0002***** |
|  | Compartment (PreTx / TVOR / Follicular) | 8.43 | **0.0006***** |  |  |
|  | Condition × Compartment | 0.64 | **0.52** |  |  |
| ILC1 | Condition (Control vs PCOS) | 0.65 | 0.52 | Neither disease status, compartment, nor their interaction significant |  |
|  | Compartment (PreTx / TVOR / Follicular) | 2.51 | 0.089 |  |  |
|  | Condition × Compartment | 0.08 | 0.91 |  |  |
| LTi-Like | Condition (Control vs PCOS) | 0.40 | 0.53 | Neither disease status, compartment, nor their interaction significant |  |
|  | Compartment (PreTx / TVOR / Follicular) | 1.29 | 0.28 |  |  |
|  | Condition × Compartment | 0.21 | 0.80 |  |  |
| CD56 bright NK cells | Condition (Control vs PCOS) | 0.51 | 0.48 | Neither disease status, compartment, nor their interaction significant |  |
|  | Compartment (PreTx / TVOR / Follicular) | 1.01 | 0.36 |  |  |
|  | Condition × Compartment | 0.71 | 0.49 |  |  |
| CD56dim NK cells | Condition (Control vs PCOS) | 0.11 | 0.73 | Effect of compartment was significant | Individual pairwise comparisons between compartments not statistically significant. |
|  | Compartment (PreTx / TVOR / Follicular) | 4.25 | **0.01*** |  |  |
|  | Condition × Compartment | 0.73 | 0.48 |  |  |
| Plasmacytoid Dendritic Cells (pDC) | Condition (Control vs PCOS) | 6.23 | **0.01*** | Disease status and compartment effect was significant | Higher in PBMC compartment  **p=0.007*** |
|  | Compartment (PreTx / TVOR / Follicular) | 7.84 | **0.0009**** |  |  |
|  | Condition × Compartment | 2.76 | 00.07 |  |  |
| Myeloid Dendritic Cells (mDC) | Condition (Control vs PCOS) | 0.85 | 0.36 | Neither disease status, compartment, nor their interaction significant |  |
|  | Compartment (PreTx / TVOR / Follicular) | 1.65 | 0.19 |  |  |
|  | Condition × Compartment | 0.085 | 0.91 |  |  |
| Intermediate Monocyes (iMO) | Condition (Control vs PCOS) | 1.11 | 0.36 | Neither disease status, compartment, nor their interaction significant |  |
|  | Compartment (PreTx / TVOR / Follicular) | 1.03 | 0.29 |  |  |
|  | Condition × Compartment | 00.068 | 0.93 |  |  |
| Classical Monocytes (cMO) | Condition (Control vs PCOS) | 3.85 | **0.04*** | Disease status and compartment effect was significant | Higher in PBMC compared to follicles (**p=0.008*).** |
|  | Compartment (PreTx / TVOR / Follicular) | 9.76 | **0.0002**** |  |  |
|  | Condition × Compartment | 0.67 | 0.51 |  |  |
| Non Classical Monocytes (ncMO) | Condition (Control vs PCOS) | 0.91 | 0.34 | Neither disease status, compartment, nor their interaction significant |  |
|  | Compartment (PreTx / TVOR / Follicular) | 1.32 | 0.27 |  |  |
|  | Condition × Compartment | 0.37 | 0.68 |  |  |
| Th17 | Condition (Control vs PCOS) | 0.49 | 0.48 | Compartment and condition*compartment interaction was significant | Post hoc pairwise comparisons between Follicular cells and PBMC compartment showed significant difference(**p=0.0003*****) |
|  | Compartment (PreTx / TVOR / Follicular) | 10.61 | **0.0001***** |  |  |
|  | Condition × Compartment | 3.89 | **0.025**** |  |  |
| CD4 T effector (eff) | Condition (Control vs PCOS) | 0.48 | 0.49 | Compartment effect is significant | Post hoc pairwise comparisons between Follicular cells and PBMC compartment showed significant difference(**p=0.02***) |
|  | Compartment (PreTx / TVOR / Follicular) | 3.52 | **0.035*** |  |  |
|  | Condition × Compartment | 1.95 | 0.15 |  |  |
| CD4 T Näive | Condition (Control vs PCOS) | 0.41 | 0.52 | Compartment effect is significant | Post hoc pairwise comparisons between Follicular cells and PBMC compartment showed significant difference(**p=0.03***) |
|  | Compartment (PreTx / TVOR / Follicular) | 3.27 | **0.04*** |  |  |
|  | Condition × Compartment | 1.84 | 0.16 |  |  |
| CD4 T Central Memory (CM) | Condition (Control vs PCOS) | 0.37 | 0.54 | Compartment effect is significant | Post hoc pairwise comparisons between Follicular cells and PBMC compartment showed significant difference(**p=0.01***) |
|  | Compartment (PreTx / TVOR / Follicular) | 4.74 | **0.02*** |  |  |
|  | Condition × Compartment | 0.02 | 0.97 |  |  |
| CD4 T Effector Memory (EM) | Condition (Control vs PCOS) | 0.001 | 0.96 | Neither disease status, compartment, nor their interaction significant |  |
|  | Compartment (PreTx / TVOR / Follicular) | 1.15 | 0.32 |  |  |
|  | Condition × Compartment | 0.34 | 0.70 |  |  |
| CD4 Exhausted | Condition (Control vs PCOS) | 0.29 | 0.58 | Effect of compartment was significant | Post hoc pairwise comparisons between Follicular cells and PBMC compartment showed significant difference(**p=0.01***) |
|  | Compartment (PreTx / TVOR / Follicular) | 6.98 | **0.001**** |  |  |
|  | Condition × Compartment | 0.80 | 0.45 |  |  |
| CD8 T Effector (eff) | Condition (Control vs PCOS) | 0.24 | 0.62 | Neither disease status, compartment, nor their interaction significant |  |
|  | Compartment (PreTx / TVOR / Follicular) | 0.27 | 0.76 |  |  |
|  | Condition × Compartment | 0.48 | 0.61 |  |  |
| CD8 active | Condition (Control vs PCOS) | 0.42 | 0.51 | Effect of compartment was significant | Post hoc pairwise comparisons between Follicular cells and PBMC compartment showed significant difference(**p=0.02***) |
|  | Compartment (PreTx / TVOR / Follicular) | 5.47 | **0.006**** |  |  |
|  | Condition × Compartment | 0.07 | 0.16 |  |  |
| CD8 Exhausted | Condition (Control vs PCOS) | 0.68 | 0.54 | Neither disease status, compartment, nor their interaction significant |  |
|  | Compartment (PreTx / TVOR / Follicular) | 0.94 | 0.3 |  |  |
|  | Condition × Compartment | 0.23 | 0.78 |  |  |
| Näive Treg cells | Condition (Control vs PCOS) | 0.17 | 0.68 | Neither disease status, compartment, nor their interaction significant |  |
|  | Compartment (PreTx / TVOR / Follicular) | 0.8 | 0.43 |  |  |
|  | Condition × Compartment | 1.18 | 0.31 |  |  |
| Memory Tregs | Condition (Control vs PCOS) | 0.011 | 0.91 | Overall effect of compartment was significant | Post hoc pairwise comparisons between Follicular cells and PBMC compartment do not reach significance  (p =0.06) |
|  | Compartment (PreTx / TVOR / Follicular) | 11.30 | **<0.001**** |  |  |
|  | Condition × Compartment | 1.09 | 0.34 |  |  |
| Activated Tregs | Condition (Control vs PCOS) | 0.56 | 0.45 | Effect of compartment was significant | Post hoc pairwise comparisons between Follicular cells and PBMC compartment showed significant difference(**p=0.005****) |
|  | Compartment (PreTx / TVOR / Follicular) | 5.32 | **0.007**** |  |  |
|  | Condition × Compartment | 0.44 | 0.64 |  |  |
| CTLA4 Treg | Condition (Control vs PCOS) | 7.03 | **0.0125*** | Effect of compartment was significant | Post hoc pairwise comparisons between Follicular cells and PBMC compartment do not reach significance  (p =0.07) |
|  | Compartment (PreTx / TVOR / Follicular) | 1.006 | 0.37 |  |  |
|  | Condition × Compartment |  |  |  |  |

**Supplementary Table S6. Number of Control and PCOS samples in each Flowcytometry experiment for tSNE and Clustering Analysis.**

| Experiment Number | Number of Patient Samples in Each Experiment | Number of Controls | Number of PCOS |
| --- | --- | --- | --- |
| 1 | 6 | 3 | 3 |
| 2 | 10 | 4 | 6 |
| 3 | 9 | 3 | 6 |
| 4 | 8 | 3 | 5 |
| Total | **33** | **13** | **20** |

**Supplementary Table S7. Cluster analysis of Innate Lymphoid Cell and NK cell. Experiment with 6 samples, 3 control and 3 PCOS**

| **Cluster #** | **Phenotype** | **Cell Annotation** |
| --- | --- | --- |
| 0 | Lin-CD45+CD45RA+ CD56+CD16+CD11b+CD117-CD127-RORyT+Tbet+CD8a | NKT |
| 1 | Lin-CD45+ CD45RA+ CD45RO+ CD56+ CD16+ CD11b+ CD117- CD127-CD94-CD141+ Tbet+ RORyT+ Eomes+ CD161+ | undefined |
| 2 | Lin-CD45+CD45RA-CD45RO+CD94-CD56-CD16-RORYT+Tbet+CD8low+ | CD8 T cell |
| 3 | Lin-CD45+ CD56-CD16-CD94-CD117+CD127-RORyT+ Tbet+ | undefined |
| 4 | Lin-CD45+CD45RA+CD11c+HLADR+CD123+RORYT + | APC |
| 5 | Lin-CD45+CD45RA+CD16+CD11c+HLADR+CD123+ | Atypical Monocyte |
| 6 | Lin-CD45+CD45RA+ CD56-CD16-CD11c-CD141+HLADR+ RORYT+ Tbet+ CD123+ | pDC |
| 7 | Lin-CD45+CD45RA+CD94-CD56-CD16-CD117-CD127-CD11b+CD11c+RORYT+ Tbet+ Eomes+ | undefined |
| 8 | Lin-CD45+CD45RA+CD94-CD56-CD16-CD11b+CD117+ CD127-RORYT+Tbet+ | undefined |
| 9 | Lin-CD45+D94-CD56-CD16-CD11b-CD117-CD127-RORYT+ | LTi like |
| 10 | Lin-CD45+CD45RA-CD94-CD56-CD16-CD11c-CD117-CD127-RORYT low,Tbet+CD123+ | pDC |
| 11 | Lin-CD45+CD45RA+CD94-CD56+CD16+CD11b+ CD117-CD127-Tbet+RORyT+Eomes low | NK cell |
| 12 | Lin-CD45+CD45RA+CD94-CD56+CD16-CD11b+CD117-CD127-RORYT +Tbet+Eomes+ | CD56+CD16- NK cells |
| 13 | Lin-CD45+CD45RA+CD94-CD56+CD16+CD11b+CD117-CD127-RORyT-Tbet-Eomes- | NK cells |
| 14 | Lin-CD45+CD45RA+CD94-CD56+CD16-CD11b+CD117-CD127-RORYT+Tbet+Eomes+ | CD56+CD16- NK cells |

Composition of clusters in PCOS vs control FF and PBMC are similar with no discernable statistical difference in cluster distributions.

**Supplementary Table S8. Cluster analysis of Innate Lymphoid Cell and NK cell. Experiment with 10 samples, 4 control and 6 PCOS.**

| **Cluster #** | **Phenotype** | **Cell Annotation** |
| --- | --- | --- |
| 0 | Lin-CD45+CD45RA+CD56-CD16-CD11c-CD141 low HLADR lowTbet+RORYT+CD123+ | pDC |
| 1 | Lin-CD45+CD45RA+CD94-CD56-CD16-CD11c+ Tbet+RORyT+Eomes- | undefined |
| 2 | Lin-CD45+CD45RA+CD94-CD56-CD16low CD11c+ CD11b low Tbet-RORyT-Eomes- | Atypical NK cells |
| 3 | Lin-CD45+CD94-CD56-CD16- CD11b+ RORYT+ CD123+ | pDC |
| 4 | Lin-CD45+CD94-CD56-CD16-CD117+CD127-RORYT+Tbet+ | undefined |
| 5 | Lin-CD45+CD45RA+CD94-CD56-CD16-CD11b+ CD117+CD127-RORYT+Tbet+ | undefined |
| 6 | Lin-CD45+CD94-CD56-CD16+ CD11b+ | CD56-CD16+ NK cells |
| 7 | Lin-CD45+CD45RA+CD94-CD56+CD16-CD11b+ R | CD56+CD16-NK cells |
| 8 | Lin-CD45+RORYT+Tbet+ | undefined |
| 9 | Lin-CD45+CD45RA+CD94-CD56hiCD16-CD11b+ RORYT+Tbet+ | CD56hiCD16-NK cells |
| 10 | Lin-CD45+CD45RA+CD94-CD56hiCD16-CD11b+ Tbet+ | CD56hiCD16-NK cells |
| 11 | Lin-CD45+CD45RA+CD94-CD56+CD16-CD11b+ RORyT+Tbet+Eomes+ | NK cells |
| 12 | Lin-CD45+CD45RA+CD45RO+CD94-CD56+ CD16+ CD11b+ CD141+CD161+RORYT+ Tbet+ | NK cells |
| 13 | Lin-CD45+CD45RA+CD94-CD56+CD16-CD11b+ RORYT+Tbet+CD8+ | NKT cell |
| 14 | Lin-CD45+Tbet+CD8a+ | CD8 T cell |

Composition of clusters in PCOS vs control FF and PBMC are similar with no discernable statistical difference in cluster distributions.

**Supplementary Table S9. Cluster analysis of Innate Lymphoid Cell and NK cell Panel. Experiment with 8 samples, 3 control and 5 PCOS**.

| **Cluster #** | **Phenotype** | **Cell Annotation** |
| --- | --- | --- |
| 0 | Lin-CD45+CD45RA+CD16-CD141+ HLADR+ Tbet+ RORYT low CD123+ | pDC |
| 1 | Lin-CD45+CD45RA+CD94-CD56-CD16+CD11b low CD11c+HLADR+Tbet+CD123+ | APC/Monocyte |
| 2 | Lin-CD45+CD11c+CD141+CD11b low HLADR+ Tbet+ | mDC |
| 3 | Lin-CD45+CD16-CD11c-HLADR-CD123+ | pDC |
| 4 | Lin-CD45+CD11c low CD11b+CD56-CD16+CD117- CD127- Tbet low | CD56-CD16+ NK cells |
| 5 | Lin-CD45+CD45RA+CD94-CD56+CD16-CD11b+ CD11c-CD117-CD127-RORyT-Tbet- | CD56+CD16-NK cells |
| 6 | Lin-CD45+CD45RA+CD94-CD56+CD16- CD11b+ CD11c-CD117-CD127-RORyT-Tbet+ | CD56+CD16-NK cells |
| 7 | Lin-CD45+ | undefined |
| 8 | Lin-CD45+CD45RA-CD94-CD56+CD16-CD11b- CD117+CD127-RORYT low,Tbet+Eomes- | undefined |
| 9 | Lin-CD45+CD45RA+CD94-CD56-CD16-CD11b+  CD117+CD127-RORYT-Tbet low, Eomes- | CD56+CD16-NK cells |
| 10 | Lin-CD45+CD45RA+CD94-CD56+CD16-CD11b+ CD117-CD127-CD8a+RORYT low,Tbet+Eomes- | NKT |
| 11 | Lin-CD45+CD45RA-CD94-CD56+CD16-CD11b+ CD117-CD127- CD8a+RORyT- Tbet low, Eomes- | NKT |
| 12 | Lin-CD45+CD45RA+CD94-CD56-CD16-CD11b+  CD117-CD127-CD8a+RORYT-Tbet+Eomes- | CD8 T cell |
| 13 | Lin-CD45+CD45RA+CD94-CD56-CD16-CD11b+ CD117-CD127-RORYT-Tbet+Eomes+ | undefined |
| 14 | Lin-CD45+CD45RA+CD94+CD56+ CD16- CD11b+  CD117+CD127-RORYT-Tbet+Eomes+ | CD56+CD16- NK cells |

Composition of clusters in PCOS vs control FF and PBMC are similar with no discernable statistical difference in cluster distributions.

**Supplementary Table S10. Cluster analysis of Antigen Presenting Cells and T cells. Experiment with 6 samples, 3 control and 3 PCOS.**

| **Cluster #** | **Phenotype** | **Cell Annotation** |
| --- | --- | --- |
| 0 | CD45+CD3+CD4+CD45RO+ | CD4 Memory T cells |
| 1 | CD45+CD3+CD4+CD45RA+CD11b+ | CD4 T cells |
| 2 | CD45+CD3+CD11b+NKG2A+ | NK cell |
| 3 | CD45+CD16+CD11b+ | NK cell |
| 4 | CD45+CD14+CD11c+CD11b+HLADR+CD16low,  LILRB1+ | Intermediate Monocyte |
| 5 | CD45+CD45RA+CD11b+CD16+HLADR+CD11c+ | Non-classical Monocyte |
| 6 | CD45+CD3+CD4+CD45RO+CD103+ | CD103+ CD4 Tcells |
| 7 | CD45+CD117+CD141+CD11b+HLADR+ | undefined |
| 8 | CD45+CD14+CD11c+CD11b+HLADR+LILRB1+ | Monocyte/Macrophage |
| 9 | CD45+CD3+CD4+CD45RA+CCR7+ | Näive CD4 T cells |
| 10 | CD45+CD45RA+CD3+CD4+CD14+CD11b+CD11+CD45RO+ CD16+ CD69+ HLADR+LILRB1+CCR7+ | undefined |
| 11 | CD45+CD45RA+CD56+CD16+CD11c+CD11b+  CD45RO+CD141+HLADR+LILRB1+CD161+ | NK cell |
| 12 | CD45+CD45RA+CD19+HLADR+CD14+CD16+CD11b+CD25+CCR6+CD103+ | undefined |
| 13 | CD45+CD19+HLADR+CD45RA+CCR 6low | B cells |
| 14 | CD45+CD45RA+HLADR+CD16low, CD123+ | pDC |
| 15 | CD45+CD45RA+CD56+CD11b+NKG2A+ | NK cell |
| 16 | CD45+CD45RA+CD56+CD16+CD11b+CD8a+ | NKT cells |
| 17 | CD45+CD3+CD45RA+CD8a+ | CD8 T cells |
| 18 | CD45+CD123+CD11b+CD16 low | undefined |
| 19 | CD45+CD3+CD8a+CD103+ | CD103+ CD8 T cells |

Composition of clusters in PCOS vs control FF and PBMC are similar with no discernable statistical difference in cluster distributions.

**Supplementary Table S11. Cluster analysis of Antigen Presenting Cells and T cells. Experiment with 10 samples, 4 control and 6 PCOS.**

| **Cluster #** | **Phenotype** | **Cell Annotation** |
| --- | --- | --- |
| 0 | CD45+CD3+CD8a+CD45RA+ | CD8 T cells |
| 1 | CD45+CD3+CD8+CD45RO+CD14+CD11b+CD11c+CD49a+LILRB1+ | undefined |
| 2 | CD45+CD14+CD11c+CD11b+HLADR+ | Monocyte/  Macrophage |
| 3 | CD45+CD45RA+CD4+CD8a+CD141+CD11c+CD45RO+HLADR+CD117+CD56+CD16+CD11b+ CCR6+CD127+LILRB1 | undefined |
| 4 | CD45+CD45RA+CD11b+11c+CD16 low LILRB1+ | undefined |
| 5 | CD45+CD14+ | Monocyte |
| 6 | CD45+CD14+CD11c+HLADR+LILRB1+ | Monocyte/  Macrophage |
| 7 | CD45+CD45RA+HLADR+LILRB1+CD123+ | pDC |
| 8 | CD45+CD3+CD8a+CD103+ | CD103+ CD8 T cells |
| 9 | CD45+CD3+CD8a+ CD45RO+ | CD8 T cells |
| 10 | CD45+CD45RA+CD117+CD11b+ | undefined |
| 11 | CD45+CD19+HLADR+ | B cell |
| 12 | CD45+CD3+CD8a+ | CD8 T cell |
| 13 | CD45+CD11b+CD16+ | Intermediate Monocyte |
| 14 | CD45+CD19+CD45RA+HLADR+CD11b+CD16+CD49a+CCR6+LILRB1 | undefined |
| 15 | CD45+CD3+CD4+CD45RO+ | CD4 T cells |
| 16 | CD45+CD3+CD4+CD45RO+CD103+ | CD103+ CD4 T cells |
| 17 | CD45+CD3+CD45RA+CD56+CD11b+ | CD3 T cells |
| 18 | CD45+CD56+CD11b+ | NK cells |
| 19 | CD45+CD3+CD45RA+CCR7 | T cells |

Composition of clusters in PCOS vs control FF and PBMC are similar with no discernable statistical difference in cluster distributions.

**Supplementary Table S12. Cluster analysis of Antigen Presenting Cells and T cells. Experiment with 8 samples, 3 control and 5 PCOS.**

| **Cluster #** | **Phenotype** | **Cell Annotation** |
| --- | --- | --- |
| 0 | CD45+CD14+CD16 low CD11c+CD11b+ HLADR+LILRB1+ | Intermediate Monocyte |
| 1 | CD45+CD45RA+CD16 low CD11c+LILRB1+ | Non-classical Monocyte |
| 2 | CD45+CD45RA+HLADR+CD123+ | pDC |
| 3 | CD45+CD45RA+CD19+HLADR+ | B cells |
| 4 | CD45+CD45RA+CD56+CD11b+CD8a low | NKT cells |
| 5 | CD45+CD14+CD11c+CD11b+HLADR+ | Monocyte |
| 6 | CD45+CD11c+CD11b+CD141+HLADR+ | Dendritic cell |
| 7 | CD45+CD45RA+CD11b+CD141+ | Dendritic cell |
| 8 | CD45+CD45RA+CD56+CD11b+NKG2A+ | NK cell |
| 9 | CD45+CD14+CD16+CD11b+ | Intermediate Monocyte |
| 10 | CD45+CD45RA+CD141+CD11c+CD56+  CD45RO+CD141+CD11b+CD4+CD117+  CCR6+CD16+CD8+LILRB1+CD49a+NKG2A+CD69+CCR7+CD161+ | undefined |
| 11 | CD45+CD45RA+CD3+CD8a+CD11b+ | CD8 T cell |
| 12 | CD45+CD3+CD4+CD45RO+ | CD4 T cell |
| 13 | CD45+CD45RA+CD3+CD4+CD45RO+  CD14+ | undefined/doublet |
| 14 | CD45+CD45RA+CD3+ | T cell |
| 15 | CD45+CD3+CD45RO+CD4+CD103+ | CD103+ CD4 T cell |
| 16 | CD45+CD3+CD45RO+CD8a+ | CD8 T cells |
| 17 | CD45+CD3+CD4+CD45RA+CCR7+ | Näive CD4 T cells |
| 18 | CD45+CD3+CD8a+CD45RA+CCR7+ | Näive CD8 T cells |
| 19 | CD45+CD3+ CD8a+CD45RO+CD103+ | CD103+ CD8 T cell |

Composition of clusters in PCOS vs control FF and PBMC are similar with no discernable statistical difference in cluster distributions.

**Supplementary Table S13. Cluster analysis of T cells. Experiment with 10 samples, 4 control and 6 PCOS**.

| **Cluster #** | **Phenotype** | **Cell Annotation** |
| --- | --- | --- |
| 1 | CD45+CD3+CD4+CD45RO+ | CD4 Memory T cells |
| 2 | CD45+CD3+CD45RO+CD11b+ | CD3 T cells |
| 3 | CD45+CD3+CD45RO+CD8a+ | CD8 MemoryT cell |
| 4 | CD45+ CD3+CD8a+CD45RA+CCR7+ | Näive CD8 T cell |
| 5 | CD45+CD3+CD4+CD45RA+CD45RO+ | CD4 T cell |
| 6 | CD45+ CD3+CD8a+CD45RO+ | CD8 Memory T cells |
| 7 | CC45+CD3+CD8a+CD45RO+HELIOS+ | CD8 Memory T cells |
| 8 | CC45+CD3+CD4+CD45RO+HELIOS+FOXP3 | Memory Treg |
| 9 | CD45+CD3+CD45RA+ | T cells |
| 10 | CD45+CD14+CD11c+CD11b+HLDR+ | Monocytes |
| 11 | CD45+CD117+CD11b+HELIOS+ | undefined |
| 12 | CD45+CD45RA+CD56+CD16-CD11b+CD94+CD117+ | NK cells |
| 13 | CD45+CD45RA+CD56+CD16-CD11b+HELIOS low | NK cells |
| 14 | CD45+CD45RO+CD3+HELIOS+ | T cells |
| 15 | CD45+CD3+CD45RA+CD56+CD11b+CD94 low+ HELIOS+ | NKT |
| 16 | CD45+CD45RA+CD11c+HLADR+CD16 low CD123+ | Dendritic cells |
| 17 | CD45+CD45RA+CD3+HELIOS+ | T cells |
| 18 | CD45+CD3+CD4+CD45RA+CCR7+ | Näive CD4 T cell |
| 19 | CD45+CD19+CD45RA+HLADR+CD69+ | B cells |
| 0 | NO FEATURES | Undefined |

Composition of clusters in PCOS vs control FF and PBMC are similar with no discernable statistical difference in cluster distributions.

**Supplementary Table S14. Cluster analysis of T cells. Experiment with 9 samples, 3 control and 6 PCOS.**

| **Cluster #** | **Phenotype** | **Cell Annotation** |
| --- | --- | --- |
| 0 | CD45+CD45RA+CD56+CD11b+CD94+HELIOS+ | NK cell |
| 1 | CD45+CD45RA+CD56+CD11b+ | NK cell |
| 2 | CD45+CD45RA+CD56+CD16+CD11b+ | NK cell |
| 3 | CD45+ CD3+CD8a+CD45RA+HELIOS+ | CD8 Effector T cell |
| 4 | CD45+ CD3+CD8a+CD45RA+ | CD8 Effector T cell |
| 5 | CD45+ CD3+CD8a+ | CD8 T cell |
| 6 | CD45+CD3+CD45RA+CD11b | undefined |
| 7 | CD45+CD16 low CD123+ | pDC |
| 8 | CD45+HLADR+CD11c+CD11b+CD141low | Dendritic Cell |
| 9 | CD45+CD45RA+CD117+CD11b+CD94+ | NK cell |
| 10 | CD45+CD3+CD4+CD45RO+ | CD4 Memory T cells |
| 11 | CD45+CD3+CD8a+HELIOS | CD8 T cells |
| 12 | CD45+CD14+CD11c+CD11b+ | Classical Monocyte |
| 13 | CD45+CD45RA+CD123+ | pDC |
| 14 | CD45+CD3+ CD4+CD8a+ CD45RA+ CD11c+ CD56+ CD16+CD11b+CD45RO+CD141+CD11b+CD117+CCR6+CCR7+CD161+ | undefined |
| 15 | CD45+Cd19+HLADR+ | B cell |
| 16 | NO FEATURES | undefined |
| 17 | CD45+CD3+CD4+CD45RA+CCR7+ | Näive CD4 T cell |
| 18 | CD45+CD3+CD4+CD45RO+HELIOS+ | CD4 Memory T cells |
| 19 | CD45+CD3+CD16+ | undefined |

Composition of clusters in PCOS vs control FF and PBMC are similar with no discernable statistical difference in cluster distributions.

**Supplementary Table S15. Cluster analysis of T cells. Experiment with 8 samples, 3 control and 5 PCOS.**

| **Cluster #** | **Phenotype** | **Cell Annotation** |
| --- | --- | --- |
| 0 | CD45+CD45RA+CD56+CD11b+CD8 low | NKT |
| 1 | CD45+CD3+CD8a+CD45RA+HELIOS+ | CD8 Effector T cells |
| 2 | CD45+CD45RA+CD3+CD8a+ | CD8 Effector T cell |
| 3 | CD45+ CD3+CD8a+ CD45RA+CCR7+ | Näive CD8 T cell |
| 4 | CD45+ CD3+CD8a+ | CD8 T cell |
| 5 | CD45+CD45RA+CD56+CD11b+CD94+ | NK cell |
| 6 | CD45+CD3+CD45RA+CD11b | undefined |
| 7 | CD45+CD14-CD16+CD11b+CD123 low | Non-classical Monocyte |
| 8 | CD45+CD45RA+CD117+CD11b+ | undefined |
| 9 | CD45+CD45RA+CD3+CD56 low CD11b+HELIOS+ | NKT |
| 10 | CD45+CD3+CD56 low HELIOS+ | NKT |
| 11 | CD45+CD14+CD11c+CD11b+HLADR+CD16 low | Classical Monocyte |
| 12 | CD45+CD45RA+CD11c+HLADR+ | Dendritic cell |
| 13 | CD45+CD45RA+CD3+HELIOS+ | T cells |
| 14 | CD45+CD117+HELIOS+ | undefined |
| 15 | CD45+CD45RA+HLADR low CD123+ | pDC |
| 16 | CD45+CD45RA+CD19+HLADR+ | B cell |
| 17 | CD45+CD45RA+CD3+CD4+CCR7+ | Näive CD4 T cell |
| 18 | CD45+CD3+CD4+ | CD4 T cells |
| 19 | CD45+CD3+CD4+HELIOS+FOXP3 | CD4 Treg cells |

Composition of clusters in PCOS vs control FF and PBMC are similar with no discernable statistical difference in cluster distributions.
